# Contrasted modifications of IgM and IgT repertoires induced by high- and low-virulent infectious pancreatic necrosis virus strains in rainbow trout (*Oncorhynchus mykiss*)

**DOI:** 10.3389/fimmu.2025.1690504

**Published:** 2026-02-04

**Authors:** Sofie Navelsaker Thommessen, Luc Jouneau, Rosario Castro, Vanessa Mhanna, Encarnita Mariotti-Ferrandiz, Hetron M. Munang´andu, Amr Ahmed Abdelrahim Gamil, Bertrand Collet, Pierre Boudinot, Oystein Evensen

**Affiliations:** 1 Faculty of Veterinary Medicine, Norwegian University of Life Sciences, Ås, Norway; 2 Université Paris-Saclay, INRAE, UVSQ, VIM, Jouy-en-Josas, France; 3 Sorbonne Université, INSERM, Immunology-Immunopathology-Immunotherapy (i3), Paris, France; 4 Institut Universitaire de France, Paris, France

**Keywords:** B cell repertoire, IPNV, teleost, viral strains, virus

## Abstract

**Introduction:**

Infectious pancreatic necrosis virus (IPNV) is a significant pathogen in salmonid aquaculture, with virulence determined mainly by amino acid residues in the VP2 capsid protein.

**Methods:**

This study investigates the modifications of rainbow trout (*Oncorhynchus mykiss*) IgM and IgT repertoire induced by two IPNV strains that differ at VP2 positions 217 and 221, specifically IPNV-TA (T_217_A_221_) and IPNV-PT (P_217_T_221_). For these studies, doubled-haploid isogenic trout were immunised with IPNV-TA or IPNV-PT, and IgHμ and IgHτ repertoires were analysed by high-throughput sequencing.

**Resutls:**

We demonstrated that IPNV-PT elicited a strong and diverse modification if the IgM repertoire in the spleen, with significant clonotypic expansions including public components, particularly within VH1, VH4, and VH8 subgroups, observed two months post-immunization. Despite this robust response, clonotype usage largely reflected a non-convergent immune profile, consisting essentially of private responses, with limited sharing across individuals. In contrast, IPNV-TA triggered only modest changes in the repertoire. Notably, the IgM response to IPNV-PT diminished by four months, with limited persistence of public clonotypes. Changes of spleen IgT repertoire remained limited.

**Discussion:**

These findings demonstrate that even minor VP2 variations can profoundly affect not only the magnitude but also the clonal architecture and composition of antiviral B cell responses, highlighting the importance of detailed, strain-specific immune profiling in guiding vaccine development for aquaculture.

## Introduction

Birnaviruses are double-stranded RNA viruses that cause severe diseases in fish, birds, and invertebrates. Infectious pancreatic necrosis virus (IPNV) is the prototypic member of the genus Aquabirnavirus and is the causative agent of a disease in juvenile salmonids, resulting in significant economic losses in the aquaculture industry (1).

IPNV virions are nonenveloped, displaying a single-shelled T = 13 icosahedral capsid made of 260 trimers of viral protein 2 (VP2) (2, 3), which is the cell-attachment protein. Specific neutralizing Abs are produced against VP2, the protein that determines virus virulence (4, 5). Two proteins are located inside the particle: VP3, which binds the genomic RNA, and VP1, the viral RNA-dependent RNA polymerase. The genome consists of two segments of dsRNA: segment A encoding the polyprotein precursor pVP2-VP4-VP3 and the non structural VP5, respectively, and segment B encoding VP1 (6, 7). As the protease VP4 cleaves its own N- and C-termini off the polyprotein, it also releases pre-VP2 and VP3 (6). VP5, also encoded by segment A, is detectable in infected cells but is not involved in the establishment of persistent infection or virulence of the virus (8).

Comparing crystal structures of VP2 capsids of IPNV and IBDV, Coulibaly et al. found that the residues important for virulence and tissue tropism are located on two different sites in the VP2 spike. The first interaction site for host receptor attachment is located at the top of the spike, and another conserved site containing an integrin-binding motif, important for internalization and uptake, is located at the base of the spike (9). The loops at the top of the spike (VP2 BC210-222, DE247-254, FG282–288 and HI314-333) are hypervariable and modulate both virulence and tissue tropism. A comparison of different IPNV strains revealed that position 221 in VP2-HVLoopBC is critical for adaptation to chinook salmon versus rainbow trout cells (CHSE-214 versus RTG-2 cell lines). T221-VP2 was required for full adaptation to CHSE cells, while A221-VP2 supported replication in RTG-2. The CHSE-adapted virus rNVI15C (T217-T221) replicated much faster and produced larger plaques than the parental strain in chinook salmon cells (rNVI15, T217-A221) (10, 11). These findings indicate that residues of the VP2-HVLoopBC are involved in virus-host cell interactions.

The virulence of IPNV is primarily determined by the VP2 residues at positions 217 and 221 (4, 5), with T217-A221 causing high mortality post-challenge in Atlantic salmon, and P217-T221 being, in principle, non-virulent. In contrast, for rainbow trout, IPNV VP2 P217-T221 is widespread, causing high mortality following experimental challenge (12). Strains belonging to genogroup 5 were found to dominate IPN outbreaks in Turkish Rainbow trout farms, in which all strains harboured the VP2 P217-T221 motif (13). Interestingly, all isolates from rainbow trout Finnish inland farms belonging to genogroup 2 or 5 harboured the same VP2 P217-T221 motif associated with lower virulence in Atlantic salmon (14). Mild clinical signs and low mortality were generally recorded in finish farms, suggesting that factors other than the presence of the virus determine the outcome of infection. The VP2 P217-T221 motif was also identified in IPNV infecting rainbow trout from Iranian (15) and Italian (16) farms. IPNV with a VP2 P217-T221 motif, therefore, appears to be widespread, or even dominant, among trout-infecting strains globally.

Importantly, the VP2 residues at positions 217, 221 and 247 within the HV loop are critical for immunogenicity (17, 18). In Atlantic salmon, the inactivated IPNV “VP2-TA” strain (T217-A221) was found to elicit the highest levels of virus neutralization (VN) titres and ELISA antibodies, whereas the “VP2-PT” strain (P217-T221) was the least immunogenic (17). Consequently, these two differences in the amino acid sequence of the VP2-HV loop were sufficient to strongly modulate the immunogenicity of the virus in its host. As previous work on the effect of different strains of IPNV infections and immunisations on the immune response has been primarily conducted in Atlantic salmon (5, 17, 19, 20), the impact of this motif on the rainbow trout B cell responses to IPNV remains unclear, and the potential implications for virus virulence in this species are still elusive.

Here we undertook a first study focusing on a comprehensive analysis of the modifications of IgHμ and IgHτ repertoires induced by two IPNV strains in rainbow trout: a “VP2-TA” strain (T217-A221) and “VP2-PT” strain (P217-T221), named below “IPNV-TA” and “IPNV-PT”, respectively. Using doubled-haploid isogenic fish, we ensured that differences in responses were not confounded by host genetic variation (21). We observed that the variation in two residues, likely involved in viral attachment to the receptor, affects the viral fingerprint in the IgHμ repertoire produced by the B cell response to immunization: IPNV-PT induced a stronger antibody (Ab) response compared to IPNV-TA. Responses also appeared to differ in terms of clonal composition, in line with the significant contribution of positions VP2 217/221 to immunogenicity. Our data provide an overview of the clonal complexity and composition of the rainbow trout B cell response to IPNV. While IPNV-PT did induce some public/shared components, the overall profile of the B cell response, particularly over time, was consistent with a non-convergent immune architecture consisting mainly of private responses.

## Materials and methods

### Fish immunization and ethical statement

**Figure 1:**
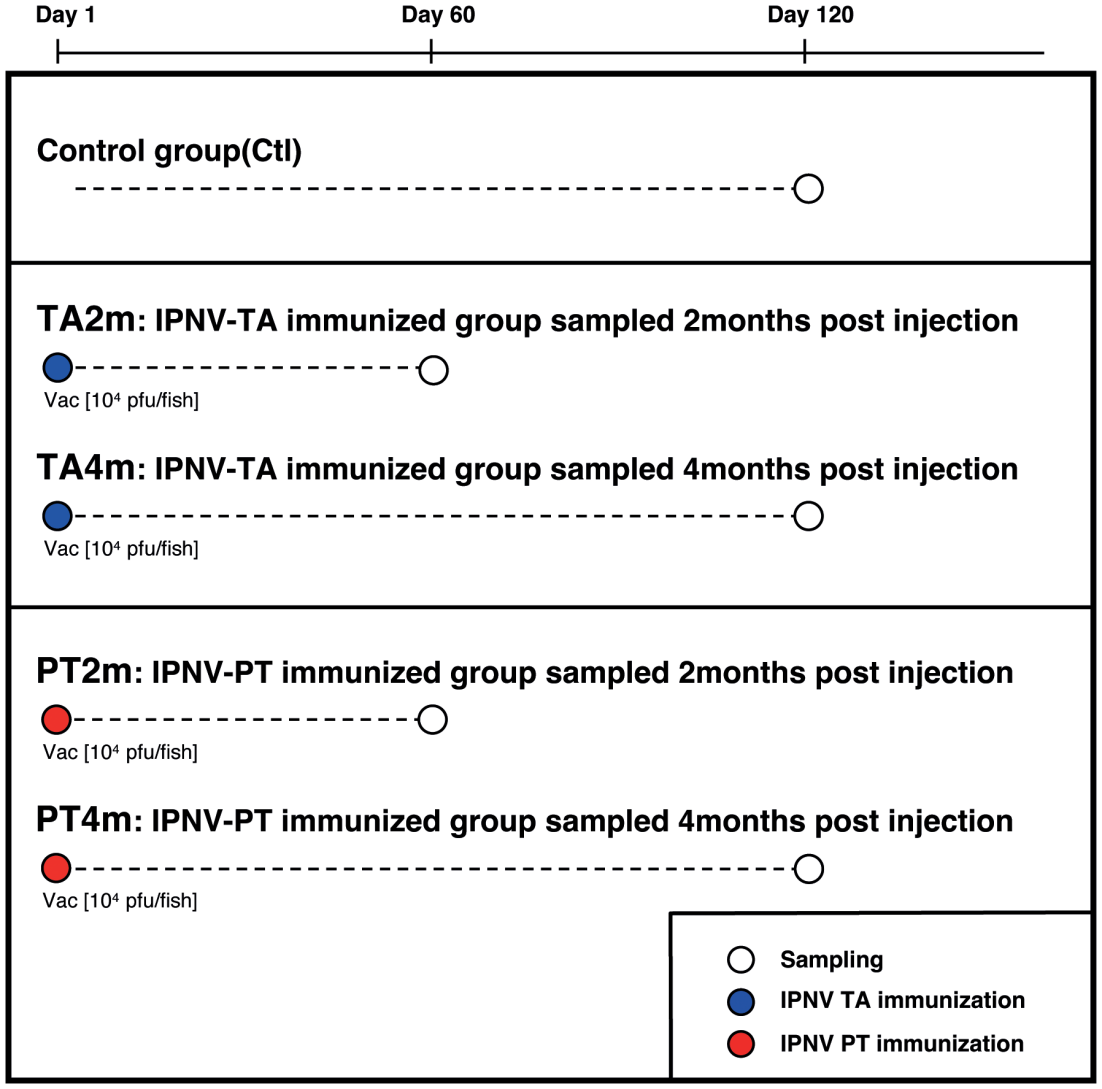
Timeline of sampling and immunisation with IPNV viruses.

Experimental groups of rainbow trout from the B57 doubled-haploid isogenic line (21) at approximately 150 g were kept in separate tanks at 16°C in the fish facilities of Institut National de la Recherche Agronomique (INRA, Jouy en Josas, France). Fish from different groups were kept in 300L tanks supplied with recirculating dechlorinated water with a flow rate of 1000 L/hour, and a photoperiod of 10:14 hours (light:dark). Fish were sacrificed through overexposure to benzocaine (100 mg/l) and subsequent destruction of the brain, followed by bleeding of the gills to remove as many erythrocytes as possible from the sampled tissues. As IPNV experimental infections typically induce mortality in rainbow trout fry, but not in subadult or adult fish, these fish were used to compare the B cell response induced by IPNV TA and PT variants. The IPNV clones IPNV-TA and PT were derived from the Norwegian Sp strain NVI015 (IPNV-TA, rNVI-015). The genetic backbone was altered using reverse genetics, focusing on AA residues 217, 221, and 247 in the VP2 capsid (GenBank accession No. AY379740 (17)), which were used for immunisations. IPNV-TA was previously found to be highly virulent and immunogenic to Atlantic salmon, whereas IPNV-PT were found to be less immunogenic to Atlantic salmon (17, 19). The two IPNV variants, IPNV-TA and IPNV-PT, are named after their VP2-capsid amino acid residues, T217 and A221, and P217 and T221, which are virulence markers in Atlantic salmon. Groups of fish received intraperitoneal injections of either IPNV-TA or IPNV-PT (1 × 10^4^ PFU/fish, same titre for both viruses) (**Figure 1**). This high dose aimed to promote a robust B cell response against the virus. The control group was sampled at day 150 post-vaccination. Trout were euthanized by overexposure to 2-phenoxyethanol diluted 1/1000. Blood was extracted and left to clot at 4°C overnight for serum extraction. The spleen was removed, fixed in RNA later (SIGMA, Aldrich), and stored at -20°C. Serum extraction was performed by centrifugation at 200 × g for 10 min. The supernatants were then collected and centrifuged at 1000 × g for 20 min, after which they were frozen at -20°C for use in titration assays. Four fish per group were sampled.

This study was conducted in accordance with the European Union guidelines for the handling of laboratory animals (http://ec.europa.eu/environment/chemicals/lab_animals/index_en.htm). All animal work at INRA was approved by the Direction of the Veterinary Services of Versailles (authorization 78-28) as well as fish facilities (authorization B78-720), and the experimental protocols were approved by the INRA institutional ethical committee “Comethea” (permit license number #15-60).

### Illumina sequencing of 5’RACE libraries

Total RNA from individual spleens was obtained in accordance with standard protocol using 1/1.2 mm ceramic beads (Mineralex, France) and TRIzol (Life Technologies, Les Ulys, France). The disruption protocol to homogenize the tissue was pulsed for 20 seconds at 10,000 rpm in a homogenizer (FastPrep 24 G5, MP Biomedicals, Santa Ana, US). Samples were then centrifuged, and the top phase, containing the RNA, was transferred to columns for further purification and DNase treatment using an RNA extraction kit (QIAGEN). Rapid amplification of cDNA 5’ ends (5’ RACE) (SMARTer RACE, Clontech) was performed to amplify all expressed IGHV genes in each sample, producing a comprehensive library of the repertoire, using 1 µg of total RNA per reaction. Libraries for Illumina deep sequencing were then prepared as described in (22). The cDNA first strand was synthetized using a primer located in the Cµ2 to target IgHμ mRNAs, excluding IgHδ, which contains a Cµ1 domain. For cDNA barcoding, the primers used for the second-strand cDNA contained a barcode of 15 random nucleotides (UID) (**Supplementary Table S1**). The resulting ds cDNA was amplified by PCR to add the Illumina adaptor sequences and a fish-specific index or barcode. The final PCR products were purified using Agencourt AMPureXP beads (Beckman Coulter, Brea, CA), and library quality was assessed using a “DNA High Sensitivity” chip on a bioanalyzer instrument (Agilent Technologies, Santa Clara, CA). Equal amounts of libraries were pooled for multiplexing, and the pools were sequenced in paired-end 2x300bp runs using a MiSeq instrument (Illumina) and the MiSeq Reagent Kit v3 (600 cycles) (Illumina), according to the manufacturer’s recommendations. This consensus-read sequencing approach, based on the incorporation of a unique UID in each cDNA molecule, allowed for the quantification of clonotype frequencies and a more accurate correction of PCR/sequencing errors.

### Annotation and data analysis

Sequences were first filtered and merged using pRESTO, and consensus sequences were computed from read pairs and annotated using IMGT/HighV-QUEST at http://www.imgt.org/, based on our previous analysis of the rainbow trout IgH locus from the Arlee strain genome assembly (23). The UID and CDR3 sequences were combined, as previously described in Magadan et al. (24), to create a unique molecular identifier (MID), which enables the correction of PCR biases and allows a better assessment of clonotype expression. Amplification bias is a major issue for producing accurate descriptions of immune repertoires using deep sequencing, and several approaches of cDNA barcoding have been developed with random UIDs to address this issue. However, because “random” UID often display biased compositions and are less diverse than expected, a given UID is often associated to several different CDR3 (hence to several different clonotypes). We offset these problems by counting MID, thus avoiding to add counts of sequences with the same UID, which do not belong to the same clonotype. A clonotype was defined for a given isotype by a triplet (V gene, J gene, CDR3 sequence), and its expression was assessed by counting the associated MID barcodes.

Global parameters of IgH repertoires were determined using the AnalyzAIRR Web-based interface (25). Repertoire diversity was assessed using the Gini index, which measures the inequality of clonotype distribution (where 0 corresponds to perfect equality, with all clonotypes represented at similar frequencies, and 1 represents perfect inequality). Differentially expressed VH genes between experimental groups were identified using the DESeq2 R package. Volcano plots were then plotted, showing log2 fold change (x-axis) and significance (−log10 adjusted p value; y-axis) of differentially expressed genes. Significantly differentially expressed clonotypes were identified when p < 0.05 and a log2 fold change threshold of > 2.

Clonotypes shared between different fish were identified and analyzed at various levels: between the top frequent clonotypes of the whole IgHμ or τ repertoire, between top frequent clonotypes expressing a given VH subgroup, or between top frequent clonotypes expressing a given IGHV gene. These approaches provided different magnifications, taking into account clonotypes detected at varying frequencies and levels of expression.

Sequence data used in this work are registered in the BioProject NCBI database with the SRA accession number PRJNA726017.

### ELISA

ELISA was used to detect IPNV specific antibodies in serum. The custom made “K95” rabbit anti-IPNV antibodies (26) diluted 1:1000 in bicarbonate buffer were used for coating 96-well ELISA plates (Thermo Fisher Scientific-Nunclon). After an overnight incubation at 4°C, the plates were washed three times with PBST (phosphate buffer saline with 0.05% tween) and blocked for 2 hours at room temperature using 250 µl/well 5% milk prepared in Everyblot Blocking buffer (Bio-Rad). The plates were then washed three times as before and incubated with either of the recombinant IPNV isolates TA and PT (5) for 2 hours at room temperature. The viruses were diluted in diluent buffer (1% milk in Everyblot Blocking buffer) and 100 µl containing about 10^5^ TCID_50_ of virus were added to each well. Subsequently, the plates were washed four times with PBST and then incubated with 100 µl/well serum samples diluted 1:80 in diluent buffer in duplicates. After 2 hours incubation, the plates were washed four times with PBST and incubated with 100 µl/well of HRP labelled mouse anti-salmonid immunoglobulin antibodies (Immunoprecise, 1 mg/ml, Victoria, Canada) diluted 1:2000 in diluent buffer for 1 hour at room temperature. Finally, the plates were washed four times with PBST and 100 µl of TMB substrate solution (Abcam, Cambridge, United Kingdom) was added to each well followed by stopping the reaction by adding equal amount 1M HCl. The incubation time with TMB was 15 min for the TA isolate and 30 min for the PT isolate. A Tecan Genios spectrophotometer (Männedorf, Switzerland) was used to measure the absorbance at 450 nm.

## Results

To analyse rainbow trout Ig responses to IPNV-TA and -PT viruses, we compared spleen IgH repertoires 2 and 4 months after immunization with each variant (**Figure 1**). The titres of anti-IPNV-TA and -PT IgM were determined by ELISA and showed that each immunisation induced a significant virus-specific antibody response (**Supplementary Figure S1**). For repertoire analysis, we used a 5’RACE sequencing approach of spleen IgH µ and τ transcripts that covered all expressed IGHV genes to assess the clonotypic composition of the B cell response to IPNV-PT and IPNV-TA. Each clonotype was defined by annotation of a V gene, a C gene, a J gene, and a CDR3 amino acid sequence, and its expression was assessed by counting a unique molecular ID (see Methods).

### IPNV-TA and IPNV-PT induce Ig responses of different strength and composition in the spleen

**Figure 2:**
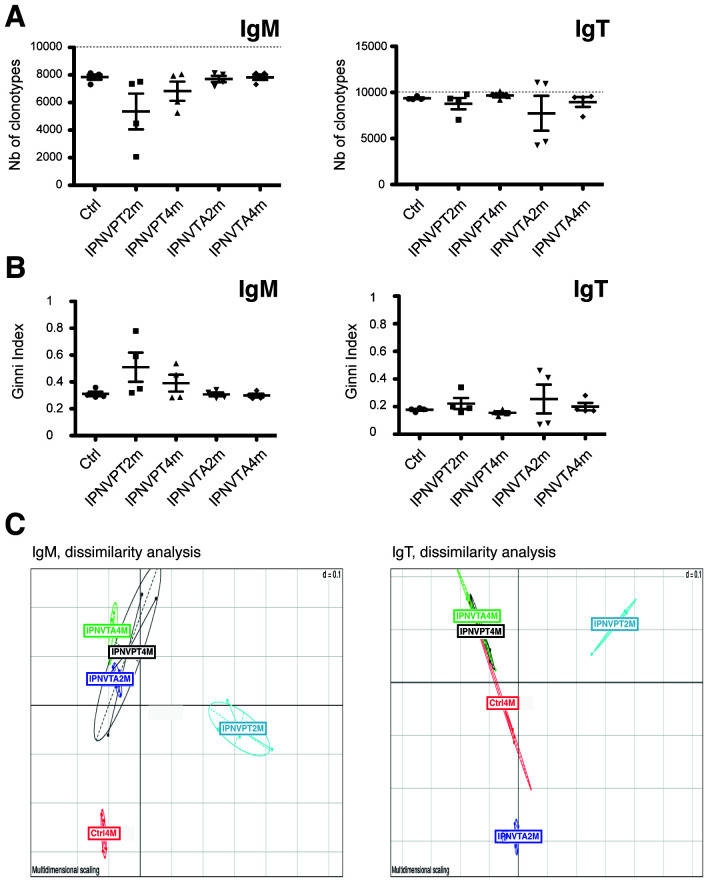
Global analysis of IgM and IgT repertoire diversity and composition. **(A)** Total number of distinct clonotypes per fish, within a subsampling of 12000 MID per fish. The number of clonotypes is higher for IgT than for IgM *within this subsampling*, indicating that the most frequent clonotypes represent a larger fraction of the repertoire for IgM. **(B)** Gini index reflecting the inequality of clonotype expression (*i.e.*, frequency within 12K MID per fish). Ctl 4 months (circle), IPNV-PT 2 months p.i. (square), IPNV-PT 4 months p.i. (up-pointing triangle), IPNV-TA 2 months p.i. (down-pointing triangle), IPNV-TA 4 months p.i. (diamond). **(C)** Dissimilarity analysis of clonotype composition of individual fish IgM and IgT repertoires. PCA based on the Jaccard distance. Although differences between groups are not statistically significant, the number of clonotypes per 12K subsampling in the control and IPNV-PT 2 months illustrates a quasi-significant trend (t-test 0.063).

A considerable decrease in diversity of the spleen IgM repertoire was observed 2 months post-immunisation with IPNV-PT (**Figure 2A**) and, to a lesser extent, also at 4 months in two of the individuals in each group. In contrast, the spleen IgT repertoire showed modest modifications in clonotype diversity after immunization with IPNV-PT; however, the diversity of IgT repertoire was reduced in two fish 2 months after immunization with IPNV-TA. These differences reveal inequalities in IgHμ clonotype frequencies, a result of the presence of fewer but larger clonotypes in responding individuals. They are also illustrated by the observed variations in the Gini index of inequality (**Figure 2B**). These analyses utilized subsampled datasets, normalized by counting the total number of clonotypes in each dataset, which enabled inter-group comparison.

A principal component analysis sorted IgHμ repertoires into three groups: the control group (Ctrl), the IPNV-PT 2 months (IPNV-PT2m) group, and all the other groups in one cluster (**Figure 2C**, left panel), consistent with the stronger response induced by IPNV-PT two months post-immunization. All immunised groups were distinct from the control. This representation was based on the “Jaccard” index, which measures the similarity and diversity of subsampled datasets without considering the frequency of clonotypes. For IgT, both IPNV-PT2m and TA2m groups stood apart (**Figure 2C**, right panel).

**Figure 3:**
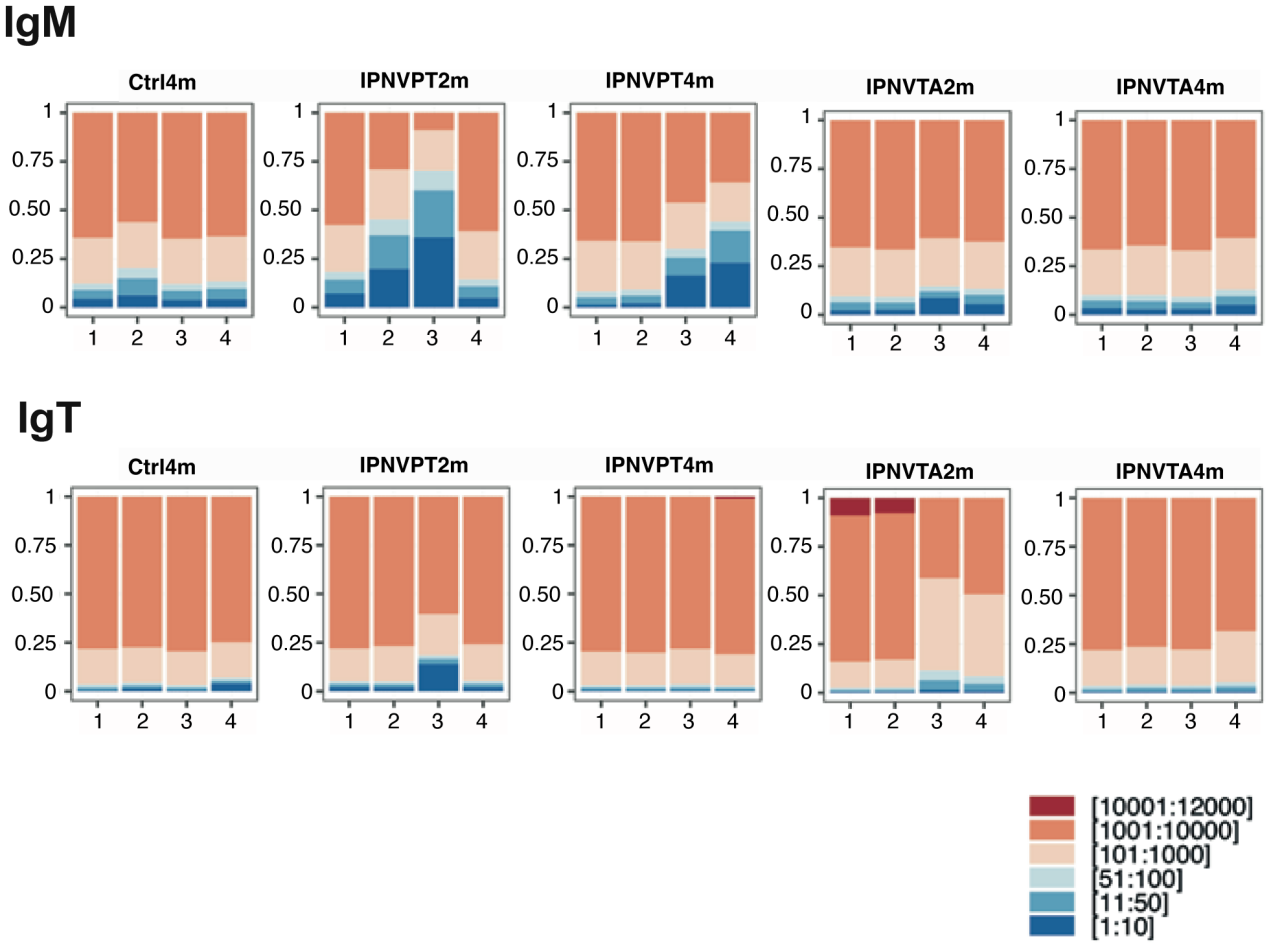
Cumulated frequency of clonotypes in each fish repertoire by ranges of rank. Within each fish dataset, clonotypes were ranked by decreasing frequency, and the cumulative frequency of clonotypes ranking 1-10, 11-100, 101-1000, 1001-10000, 10001–12000 was represented. Ranges of ranks (rank = 1 being the most frequent clonotype) are color-coded as indicated at the bottom. The number of each fish from the experimental groups is indicated on the x axis of each panel.

We then represented the proportion of clonotypes in each fish, classified by rank ranges. To this aim, clonotypes were ranked by decreasing frequency within each fish dataset, and the cumulative frequency of clonotypes ranking 1-10, 11–100 etc was represented (**Figure 3**). These graphs reflect the strength of the Ig response and visualize modifications of every individual’s repertoire after immunization. Again, the strongest expansions were detected for IgM in fish immunised with IPNV-PT. The classification of clonotype frequency by decreasing rank is another way to visualize these differences (**Supplementary Figure S2**). Consistently, the number of highly frequent clonotypes was significantly greater in fish immunised with IPNV-PT compared to fish immunised with IPNV-TA or controls (**Supplementary Figure S2**). The top 5 IgM clonotypes were 10 to 5 times more frequent in IPNV-PT2m (and also in IPNV-PT4m) than in controls (**Supplementary Figure S2A**). Another striking finding is that the expression of IgM clonotypes ranked between [10:1000] in IPNV-PT2m was much higher compared to the other groups, including IPNV-PT4m, suggesting a substantial modification of the repertoire in this group. The IgT profiles differ markedly from the IgM profiles. The number of frequent IgT clonotypes (>100 counts) in IPNV-PT immunised fish was lower than for IgM. Additionally, top IgT clonotypes were generally expressed at significantly lower levels than those of IgM, and the IPNV-PT2m group was the only immunised group in which the top 5 clonotypes were more frequent than in controls (**Supplementary Figure S2B**).

These data show that the IgM response induced by the IPNV-PT variant is stronger compared to IPNV-TA and that slight modifications of the IgT spleen repertoire can be detected for both variants, mainly two months post-immunisation. As suggested by the diversity analysis, frequency distributions show that the level of response can differ significantly from fish to fish within a group, even though they were all inoculated with the same number of PFU.

### Differential analysis of expression identifies VH subgroups and V-J gene combinations involved in response to IPNV-PT

**Figure 4:**
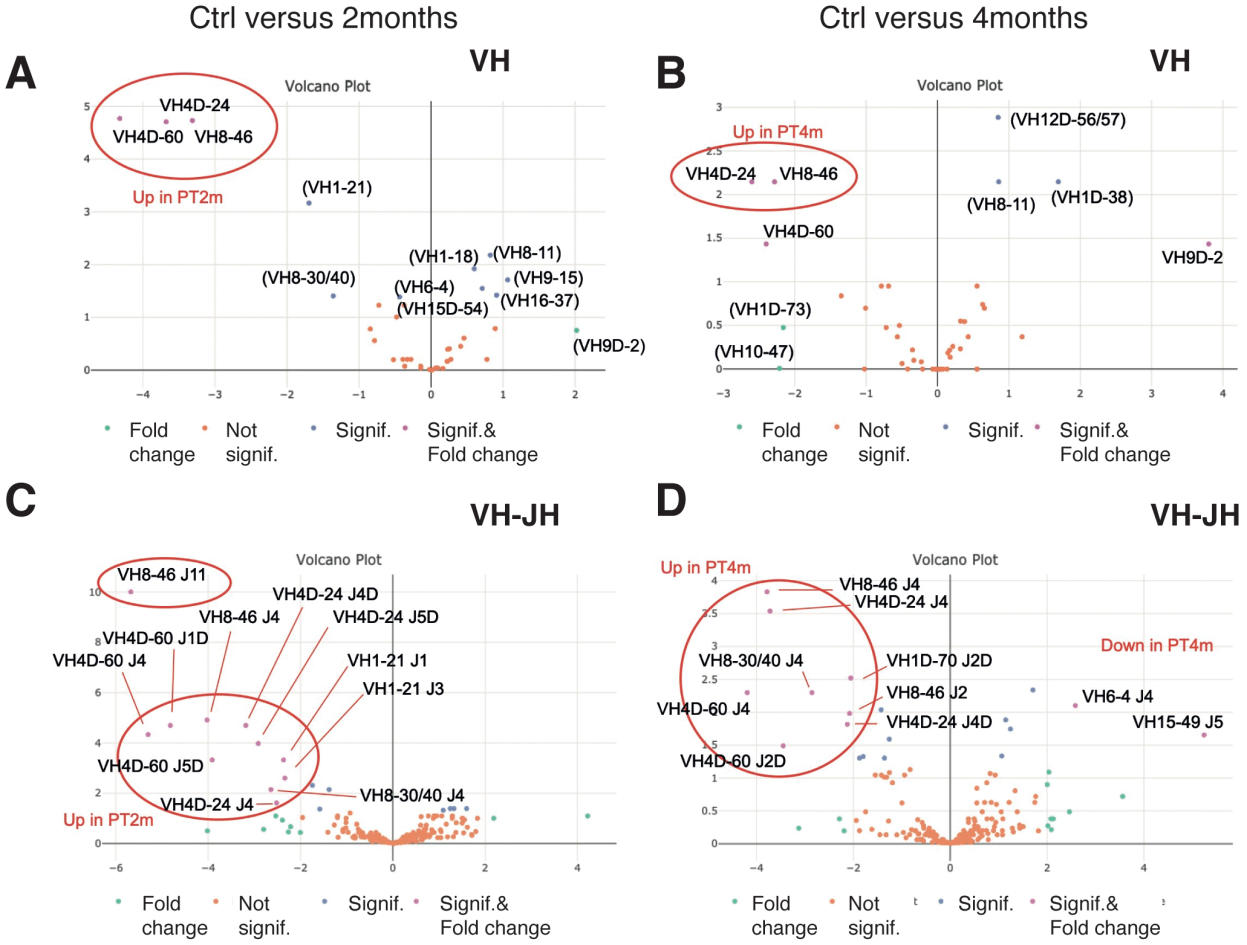
Differential analysis of expression of IGH genes 2 months **(A)** or 4 months **(B)** after immunization with the PT virus (for IgHμ). [X= log (FC); Y= log(*p*-value)]. VH (top panels) and VH-JH combinations (bottom panels) were analyzed. Colour codes are given at the bottom of each panel and refer to fold change or to significant *p*-value (5%). Relevant VH/JH have been indicated between () if not with a significant *p* value, but |FC| > 2. Several annotations combined VH and JH from different IGH loci, which was most likely due to high error frequency in JH annotation (JH identification is based on short sequences that are similar to each other, leading to a higher risk of mis-annotation). This analysis is based on data from four fish per group (subsampling of 12000 MID per fish).

The IGHV gene usage in the spleen IgHμ repertoire was relatively homogeneous across control fish. For IgM, the five most expressed IGHV genes (out of 46 annotated by IMGT V-QUEST) in the control group were IGHV6-4, IGHV9-45, IGHV1-2, IGHV1-18, and IGHV1-21, which represented approximately 50% of the total expressed repertoire in controls (**Supplementary Table S2**). To understand how the response induced by IPNV-PT modified the VH distribution, we then performed a quantitative analysis of expression in both control and immunised groups (**Figure 4**).

For IgM, differential analysis of IGHV expression revealed that IGHV4D-24, IGHV4D-60, and IGHV8–46 were significantly more expressed in individuals infected with IPNV-PT than in controls. VH1–21 was also more frequent in immunised fish, but the difference was not significant (**Figures 4A, B**). Similar analyses for IGHV/IGHJ combinations refined these results (**Figures 4C, D**), pointing to several combinations, including IGHV4D-24/J4D and J5D, IGHV4D-60/J1D, IGHV8-46/J4 and J11, and IGHV1-21/J1 and J3. The expression of an IGHV8 gene, annotated IGHV8-30/40 (**Figure 4D**), combined with IGHJ4, was also significantly upregulated in fish immunised with IPNV-PT. Overall, VH and VH-JH analyses indicate that gene usage shifts are consistent between the IPNV-PT2m and IPNV-PT4m groups, supporting the notion that these shifts are associated with the antiviral response. No significant differences were found when comparing controls and fish immunised with IPNV-TA. This was expected because previous analyses had shown that the repertoire was not strongly modified overall (**Supplementary Figure S3**). For IgT, differential analysis did not identify any significant shift either (data not shown, **Supplementary Figure S4**).

In addition to the genes identified by the differential analysis, the IGHV expression heatmaps (**Supplementary Figure S5**, upper panel) showed that IGHV2–8 and IGHV8-30/40 were upregulated in the IgM repertoire in some individuals, but not all immunised with IPNV-PT or TA. For IgT, we observed that IGHV1-36*01, IGHV6-35*01, and IGHV8-19*01 may also be used in responses to both IPNV-PT and TA (**Supplementary Figure S5**, lower panel).

Taken together, these data identify VH1, VH4D and VH8 as three main VH subgroups involved in the response to IPNV-PT in rainbow trout.

### Does the rainbow trout response to IPNV lead to the expansion of public IgHμ clonotypes?

Differential analysis of expression of VH subgroup or IGHV/J gene combinations between infected and control fish provided a statistical hint of their implication in the response but did not identify responding clonotypes. Indeed, we cannot entirely exclude that a top frequent clonotype in an infected fish had been expanded before immunization in several fish, for example, in response to another antigen. A possible approach to identifying responding clonotypes is to focus on highly shared or public clonotypes with high expression levels, *i.e.*, to look for sequences that are consistently expanded in (almost) all fish immunised with a given virus, but not in controls.

**Figure 5:**
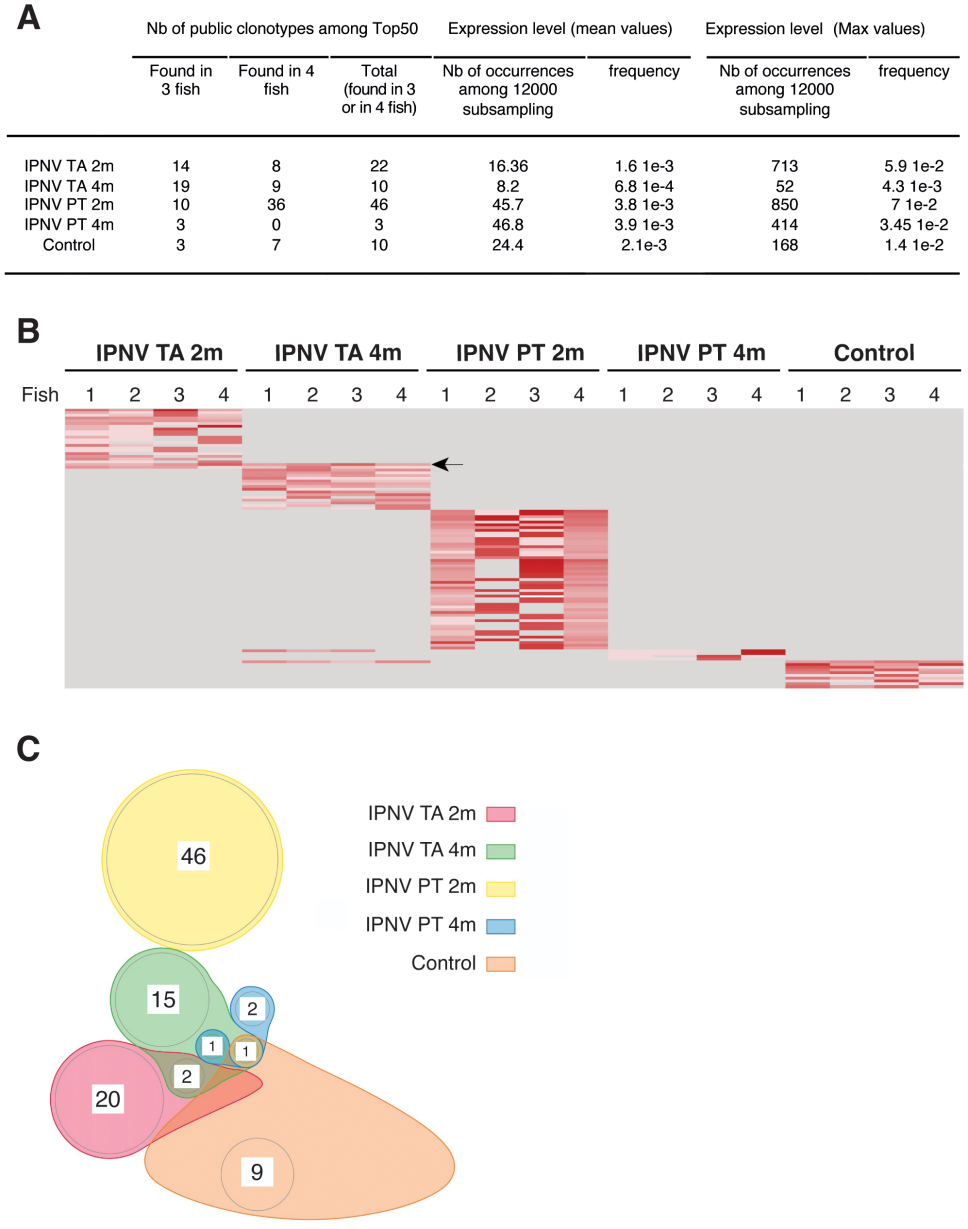
Highly shared clonotypes within each experimental group. **(A)** Numbers and frequency of highly shared clonotypes among the 50 most frequent clonotypes in each experimental group. Subsamples of 12000 clonotypes per fish were analyzed. Highly shared clonotypes are defined as clonotypes detected in subsamples of 3 or 4 individuals out of the 4 within a group. To identify the 50 most frequent clonotypes in each experimental group, all clonotypes across the four fish of each group were ranked based on their frequency. **(B)** Heatmap of frequency of these highly shared clonotypes across all fish **(C)** Venn diagram showing that most of the highly shared clonotypes were detected in only one group.

We, therefore, ranked the most frequent clonotypes in each experimental group based on the cumulated expression of all clonotypes across the four fish of each group. Among the 50 most frequent clonotypes within a group, we then identified highly shared clonotypes present in 3 or 4 fish within each group (**Figure 5A**). Strikingly, 46 out of the 50 most frequent clonotypes in the IPNV PT 2m group were shared across all individuals in this group, in contrast to only 10 clonotypes in the control group and approximately 20 in fish immunised with the IPNV TA virus. Surprisingly, only 3 clonotypes were highly shared within the IPNV PT 4m group, suggesting a profound change in IgHμ repertoire structure in two months. This finding was entirely consistent with our global analysis of the repertoire, which identified the IPNV PT 2m as the only group showing a strong response. Importantly, none of the highly shared clonotypes observed in the IPNV PT 2m or 4m groups were common to both, suggesting the absence of a persistent public clonotype response to this virus (**Figures 5B, C**), unlike what has been previously reported for VHSV in the spleen and head kidney of rainbow trout. Minimal overlap was observed between groups, and one of the two clonotypes found in both IPNV TA 2m and 4m groups (IGHV6-4, IGHJ4, CDR3=ARLYYRFDY; see arrow in **Figure 5B**) was characterized as a highly frequent clonotype in non-immunised fish in our previous work (22).

Thus, the response to IPNV PT involved convergent and shared clonotypes, which are likely directed against viral epitopes. However, we could not find a strong and stable public arm in this response (*i.e.*, present in IPNV PT-immunised fish 2 and 4 months post-immunisation), and no common component was found with the repertoire of fish immunised with IPNV TA either.

### Multiple VH subgroups are involved in the response against IPNV-PT and reflect different compositions and kinetics of response against IPNV-TA

**Figure 6:**
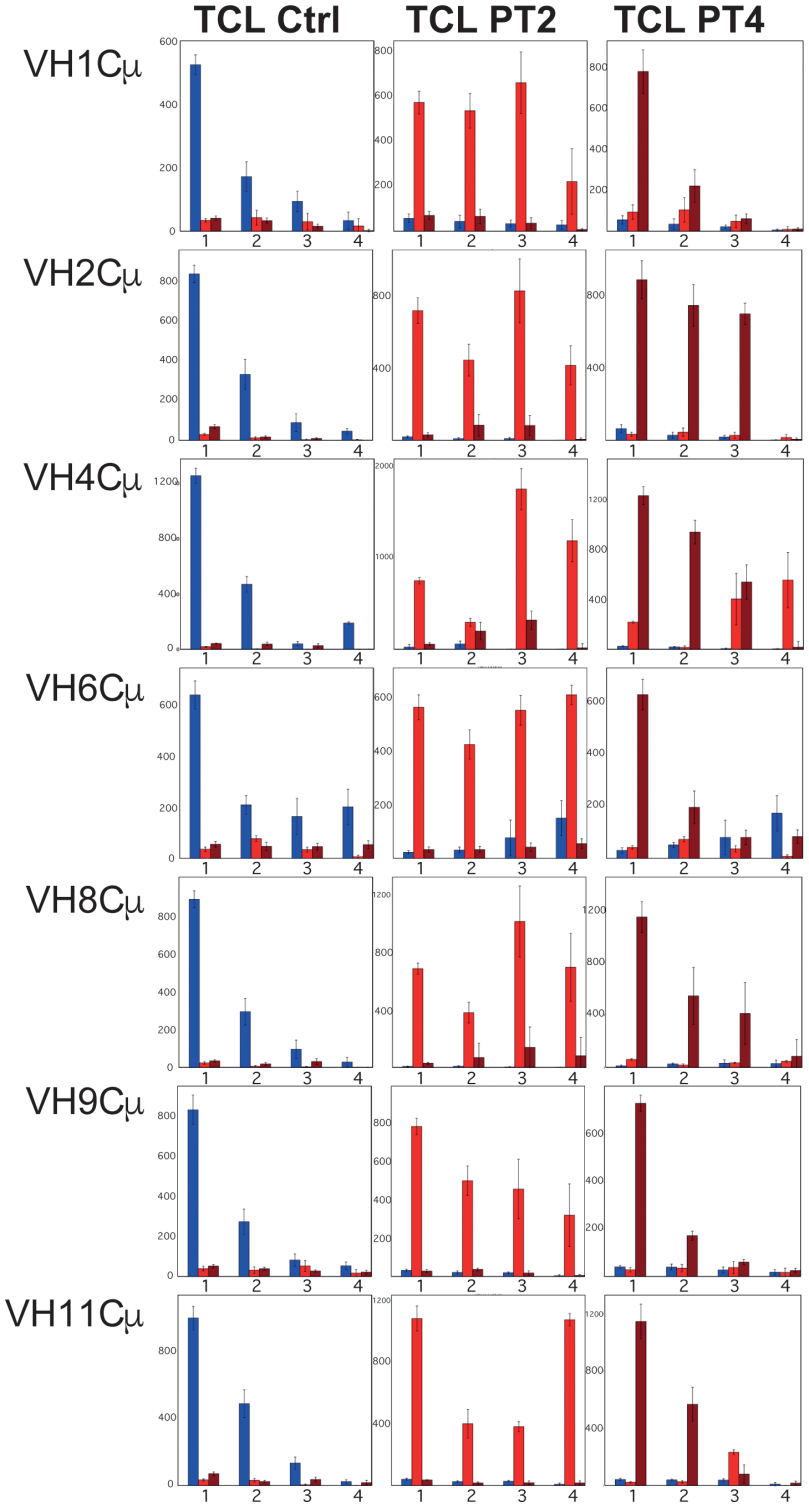
Highly shared clonotypes within IGHV subgroups. Cumulative expression of IgM Top50 clonotypes shared by n individuals (n=1, 2, 3 or 4) within the control group (Ctrl) and groups immunised with IPNV-PT sampled after 2 months (PT2) or 4 months (PT4). Lists of Top50 clonotypes (TCL) are defined here *for a given VH subgroup* within each analyzed experimental group (Ctrl, PT2 or PT4), as the nonredundant union of the lists of Top50 clonotypes expressing this VH subgroup, computed from the 4 fish belonging to each experimental group. The cumulative expression and sharing of clonotypes from the TCL_Ctrl reference list are represented in the left panels. In contrast, clonotypes from the TCL_PT2 or TCL_PT4 lists are analyzed in the middle and right panels, respectively. Bar plots show the total expression and sharing of the clonotypes from TCLs of each fish group, either among Ctrl (in blue), among PT2 (in red) or PT4 (in dark red): in each small panel, bars noted 1, 2, 3 and 4 represent the cumulative expression of clonotypes found in only one fish, in 2, in 3, or 4 fish, respectively. Bars are computed from the average values corresponding to top clonotypes found in 1–4 fish over 10 subsamplings of 10,000.

To identify shared components of the response using a more sensitive approach, we then focused on the 50 most expressed clonotypes identified in each fish *for each VH subgroup* (“Top 50” clonotypes). We analysed their presence across fish within the same experimental group or across different groups. The sharing level between different fish was represented in a bar plot as the cumulative frequency of Top50 IgHμ clonotypes *present in n individuals* (n = 1, 2, 3, 4) within a given group (**Figure 6**). We examined the sharing of clonotypes between control and IPNV-immunised groups, comparing PT2-PT4 or TA2-TA4 for IgM and IgT (**Figure 6**, **Supplementary Figures S6A–C**).

#### Top clonotype sharing analysis in IPNV-PT immunised fish per IGHV subgroup: persistent IgM response for VH2 and VH4 subgroups

In **Figure 6**, plots in the left column (TCL-Ctrl) show that the Top50 clonotypes from the control fish (blue bars) were poorly shared across the control fish. These clonotypes were either absent or found at low frequency in the immunised groups (left plots, red bars (IPNV-PT2), and dark red bars (IPNV-PT4)).

In contrast, a significant fraction of IPNV-PT2 Top50 clonotypes (TCL_PT2, bar plots of the middle column) were shared within this group (red bars) for all main VH subgroups. This trend was especially pronounced for VH4 and VH8; higher cumulative frequencies were also observed compared to the controls for these two VH, indicating substantial expansions. The top 50 clonotypes from the IPNV-PT2 group were expressed at very low frequencies in control fish (blue bars, middle column). More strikingly, while we compared responses in isogenic fish sharing the same genetic background, these clonotypes were also poorly expressed in IPNV-PT4 fish studied 4 months post-immunization (middle panels, dark red bars). This indicated a major shift in clonotype expression within this time frame: repertoire modifications detected 2 months post-immunisation were not observed in fish sampled 2 months later.

The top 50 clonotypes from the IPNV-PT4 group (bar plots in the right column) were poorly shared among most VH subgroups (dark red bars), indicating that the level of sharing had returned to normal, except for the VH2 and VH4 subgroups. Remarkably, for VH4, Top clonotypes from the IPNV-PT group were also well-expressed and highly shared in fish analyzed 2 months post-immunization (VH4, right panel, red bars) but not in controls. Thus, while they were not among the IPNV-PT2 Top50 clonotypes, they were already expanded in >=3 fish compared to controls. This observation indicates persistence of expanded clonotypes over 4 months after immunization and further supports VH4 involvement in the B cell response against IPNV-PT virus.

These results show a wide IgM response against IPNV-PT 2 months post-immunization, which has largely disappeared 2 months later. For IgT VH1, VH4, and VH8, the top clonotypes were highly shared 2 months post-immunisation (suggesting that some response had occurred) but not at 4 months (**Supplementary Figure S6A**).

#### Top clonotype sharing analysis in IPNV-TA immunised fish reveals different VH responses and kinetics

For IgM, an increase in clonotype sharing occurred only for VH8, both in the IPNV-TA2 and IPNV-TA4 groups, and to a lesser extent also for VH2 and VH6, but only in the IPNV-TA4 group. These observations showed that while immunisation with IPNV TA did not significantly alter the global parameters of the IgHμ repertoire, it induced more subtle modifications, indicating that an immune response had occurred. The main VH subgroups detected in response to IPNV-TA and -PT were distinct, which may also be related to different response kinetics. For IgT, the shift was more pronounced (although primarily limited to sharing by 2 or 3 fish), affecting the VH1, 4, 6, 8, and 9 subgroups. This occurred only in the IPNV-TA2 group, while the profiles of IPNV-TA4 fish were nearly identical to those of the controls.

Overall, these data indicate that rainbow trout response to immunisations with IPNV-TA and -PT viruses is quantitatively and qualitatively different, exhibiting distinct sets of shared clonotypes at varying rates and intensities. This is also well-illustrated by a similar sharing analysis comparing IPNV-PT and IPNV-TA immunised groups at each time point (**Supplementary Figure S7**).

### Detailed insights into responses induced by IPNV from Top clonotype sharing analysis at the IGVH gene level

To identify clonotypes potentially involved in shared or public responses, we complemented the previous analyses with a similar sharing approach *at the IGHV gene level*, focusing on the 50 most expressed clonotypes identified in each fish for each IGHV gene.

**Figure 7:**
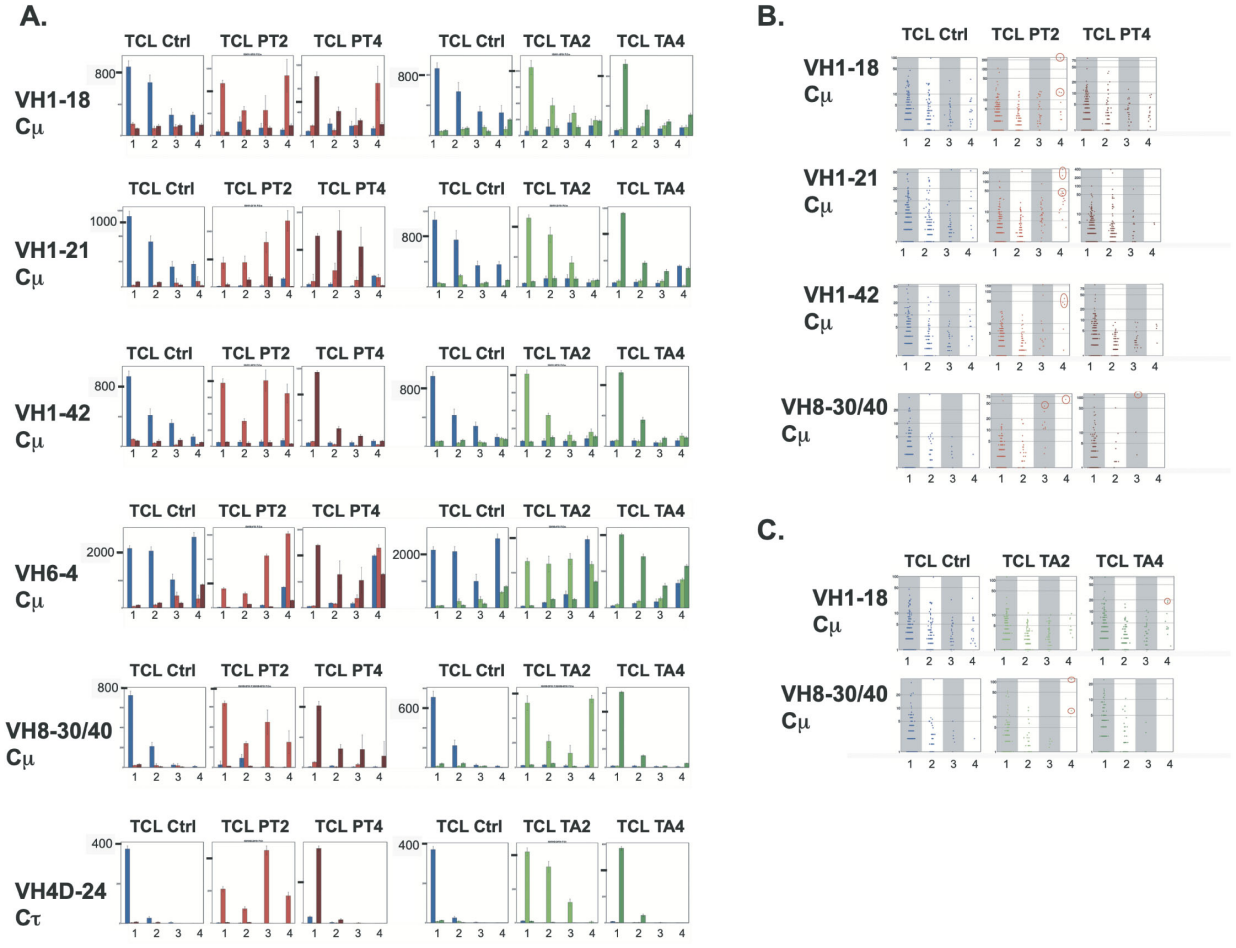
Highly shared clonotypes at the IGHV gene level. **(A)** Left section. Cumulative expression of IgM Top50 clonotypes shared by n individuals (n=1, 2, 3 or 4) within the control group (Ctrl) and the groups immunised with IPNV-PT sampled after 2 months (PT2) or 4 months (PT4). Lists of Top50 clonotypes (TCL) are defined here *for a given IGHV gene* within each analyzed experimental group (Ctrl, PT2 or PT4), as the nonredundant union of the lists of Top50 clonotypes expressing this IGHV gene, computed from the 4 fish belonging to each experimental group. The cumulative expression and sharing of clonotypes from the TCL_Ctrl reference list are represented in the left panels. In contrast, clonotypes from the TCL_PT2 or TCL_PT4 lists are analyzed in the middle and right panels, respectively. Bar plots show the total expression and sharing of the TCL clonotypes of each fish group, either among Ctrl (in blue), among PT2 (in red) or PT4 (in dark red): in each small panel, bars noted 1, 2, 3 and 4 represent the cumulative expression of clonotypes found in only one fish or 2, 3, or 4 fish, respectively. Bars are computed from the average values corresponding to top clonotypes found in 1–4 fish over 10 subsamplings of 10,000 - Right section. A similar analysis is shown for controls (blue bars), TA2 (green), and TA4 (dark green). The numbers on the Y-axis of TCL_Ctrl panels correspond to markers on the Y axis of TCL_PT or TLC_TA panels. **(B, C)** Alternative representation of sharing analysis of Top50 clonotypes from Ctrl, PT2 and PT4 TCLs (as defined above) in **(B)** or Ctrl, TA2 and TA4 TCLs in **(C)**, showing clonotype expression levels. Selected IGHV are represented. The data are from a given subsampling of 10000 per individual.

Two months after immunisation with IPNV PT, when the IgM response was at its maximum, relevant shared clonotypes were identified for multiple IGHV genes, including IGHV1-18, IGHV1-21, IGHV1-42, and IGHV4D.24, IGHV4D.60, IGHV8.5, IGHV8.19, IGHV8.30/40, IGHV8.46 and IGHV9.23 (**Figure 7** and **Supplementary Figure S8A1**). Top clonotypes expressed and shared at different times (2 or 4 months) post-immunisation were distinct, again supporting that the main components of the response between these time points differed. The few clonotypes shared by most fish in both PT-2 and PT-4 groups were present at very low frequencies at 4 months post-immunization. No substantial increase in top clonotype sharing was observed at this level of analysis, two months after IPNV-TA immunisation (except possibly for VH8-30/40), following the weaker response described above (**Figure 7**, **Supplementary Figure S8B1**).

For IgT, many IGHV genes showed an increase in sharing within the PT2 group, but this pattern did not persist in the PT4 group (**Supplementary Figure S8A2**). No modification was found after immunization with IPNV-TA (**Supplementary Figure S8B2**).

Comparing IgM responses induced by IPNV-TA and IPNV-PT, we found that the Top 50 clonotypes were generally distinct (**Supplementary Figure S8C1**), confirming the trend observed above.

We then took a closer look at individual clonotypes involved in the highly shared/public IgM response. For the selected IGHV, **Figures 7B, C** show the average expression level of clonotypes present in 1, 2, 3, or 4 fish in each group (across the individuals in which they are expressed). Based on our analysis at different levels, we identified public clonotypes among TCL_PT and _TCL_TA lists, of which sequences and frequency are presented in **Table 1**. Interestingly, we observed that several distinct clonotypes that were highly expressed and shared by all individuals of the PT2 groups displayed highly similar sequences: they differed either by the selected JH (like the two first clonotypes in **Table 1**) or by a conservative substitution in the CDR3 (like the IGHV1–21 clonotypes with ARGRIYFDY and ARGRLYFDY CDR3s). This convergence is a typical feature of public responses.

**Table 1:** Expression of clonotypes highly shared within groups of IPNV-immunised fish.

	IgHV	IgHJ	CDR3 (aa)	Mean 1^st^ fish	Mean 2^nd^ fish	Mean 3^rd^ fish	Mean 4^th^ fish	Mean (all group)
IGHV1-18								
IPNV-PT-2months	IGHV1-18*01	IGHJ2D	ARYYGRAFDY^3^	1^1^	837	16	55	227^2^
	IGHV1-18*01	IGHJ3,5D	ARYYGRAFDY^3^	58	2	3	3	17
	IGHV1-18*01	IGHJ2,4D	ARYTDYYFDY	40	1	1	26	17
IPNV-TA-4months	IGHV1-18*01	IGHJ2,4D	ARYITDYFDY	20	33	3	17	18
	IGHV1-18*01	IGHJ2D	ARYTGYAFDY	3	11	12	41	17
IGHV1-21								
IPNV-PT-2months	IGHV1-21*01	IGHJ3,5D	ARDRRNGAFDY	1	853	115	38	252
	IGHV1-21*01	IGHJ2,4D	ARGRLYFDY^3^	544	4	58	25	158
	IGHV1-21*01	IGHJ2,4D	ARGRIYFDY^3^	98	1	10	4	28
	IGHV1-21*01	IGHJ2D	ARDLRGNAFDY	1	19	165	9	48
	IGHV1-21*01	IGHJ1,1D	ARARYYFDY	138	2	14	5	40
	IGHV1-21*01	IGHJ3,5D	ARDPRRDAFDY	123	1	13	6	37
	IGHV1-21*01	IGHJ2,4D	ARAPDNYFDY	114	1	13	13	37
IGHV1-42								
IPNV-PT-2months	IGHV1-42*01	IGHJ2D	ARGRITAFAFDY	1	224	7	3	59
	IGHV1-42*01	IGHJ3,5D	ARATFNAFDY	168	3	8	6	46
	IGHV1-42*01	IGHJ3,5D	ARYTDNAFDY	145	1	7	83	59
	IGHV1-42*01	IGHJ4	ARQNNYRFDY	1	153	4	3	40
IGHV4D-24								
IPNV-PT-2months	IGHV4D-24	IGHJ1,1D	ARQASARYFDY	3	134	61	15	53
IGHV4D-60								
IPNV-PT-2months	IGHV4D-60	IGHJ2,4D	ARKAGYYYFDY	93	1	36	19	37
	IGHV4D-60	IGHJ4	ARRGNYRFDY	108	2	37	18	41
IGHV8-30/40								
IPNV-PT-2months	IGHV8-30/40*01	IGHJ1,1D	ARGRITAVRYFDY	227	12	2	6	62
IPNV-PT-4months	IGHV8-30/40*01	IGHJ4	AKGPNNGCRFDY	5	2	364	0.4	93
IPNV-TA-2months	IGHV8-30/40*01	IGHJ3,5D	ARGGFSFDY	58	36	371	12	119
	IGHV8-30/40*01	IGHJ3,5D	ARDKNNGDFAFDY	6	5	56	1	17

^1^ Average expression of clonotypes within a subsampling of 10000 sequences expressing a given IGHV gene (nb of subsampled MID are indicated for each VH gene) ^2^ Mean of the four average values computed for each fish of a group ^3^ Sequences highly similar found in distinct highly shared clonotypes (*i.e*., “convergent”), a hallmark of public response.

Our data, therefore, identify IGHV genes and rearrangements implicated in a (modest) trout public response to IPNV-PT. However, we found no large clonotypes that were expanded in both IPNV-PT and IPNV-TA immunised fish, indicating that B cell responses against these variants do not share public components.

## Discussion

In this work, we compared rainbow trout B cell responses elicited by two IPNV strains differing only by two residues critical for the immunogenicity of the virus and for its virulence. IPNV-PT viruses were of particular interest in this species because they appear to be prominent in IPNV outbreaks in rainbow trout farms, also associated with high mortality following experimental challenge (12). As the IPNV-TA variant induces high antibody and neutralizing titres in Atlantic salmon—while also being the most virulent in this species—and the less virulent IPNV-PT variant elicits a weaker B cell response, it was of particular interest to compare the responses to these two IPNV variants in rainbow trout, where their relative virulence is reversed. Identifying the immunological differences between trout and salmon, the B cell response to these viruses may be relevant to designing optimised, species-specific vaccine strategies against IPNV infection.

### IPNV-PT induces stronger modification of the IgM repertoire than IPNV-TA in rainbow trout

While both IPNV-TA and -PT induced significant titres of virus-specific IgM, our study demonstrates that only immunisation with live IPN virus harbouring the VP2 P_217_-T_221_ motif resulted in marked modifications of the spleen B cell repertoire for several weeks. In contrast, immunisation with the IPNV_TA variant resulted in only modest perturbations of the spleen IgH repertoire, which may rather affect IgHτ. These observations raise several issues. The possibility that both strains, IPNV_PT and IPNV_TA, can persist in the HK for a longer period (up to 2 months or even longer) cannot be fully excluded. Therefore, it would be of interest to monitor the virus load after immunizations with IPNV PT and TA strains, which was not conducted in this study. While IPNV_TA is more virulent in Atlantic salmon, the high frequency of IPNV outbreaks with IPNV_PT in trout farms suggests that the VP2 PT motif may be an adaptation to rainbow trout. Further studies should address this point, also comparing the susceptibility of young fish to the two variants.

It has been shown that two substitutions in VP2 at positions 217 and 221 can cause a dramatic shift in IPNV immunogenicity and virulence in Atlantic salmon (5, 17). Here, we extend these observations to study the impact of these mutations on the B cell response in rainbow trout. Surprisingly, differences in IgH repertoire changes induced by IPNV TA and PT were both quantitative (degree of repertoire perturbation and kinetics) and qualitative (absence of shared clonotypic expansions), likely excluding the possibility that they result only from a less efficient stimulation of specific B cells.

In fact, VP2 mutations at amino acid positions 217 and 221 have a significant impact on viral replication and entry and the earliest stage of infection (10, 11). There are many examples of viral variants in which only one or two modifications lead to significant differences in replication efficiency, tropism, or virulence. For example, a A82V mutation in the glycoprotein of the Ebola virus led to heightened intrinsic ability to infect primate cells, including human dendritic cells, and was associated with increased disease severity (27). One or two differences in the NS3 helicase of West Nile virus or in the NS2B of Zika virus induces significant changes in replication rates (28, 29). Thus, such mutations may modify both the abundance and spatial distribution of viral antigens, thereby influencing the induction of B cell responses.

Differences in key epitopes may also impact antigen presentation, but using isogenic rainbow trout with a defined genetic MHC background, excludes that such mechanisms *per se* would explain the differences between IPNV-TA and -PT.

As positions 217/221 are located in the immunogenic hypervariable loop of VP2 and directly modulate the strength and quality of the B cell response in Atlantic salmon (17), they are also likely to modulate critical epitopes. Such effects would provide a plausible explanation for the differences between clonotypic responses in rainbow trout. Location of position of 217/221 in IPNV-TA and IPNV-PT VP2 molecular models illustrates the possible influence of these substitutions on the immunogenic loop of the protein (**Supplementary Figure S8**). Such a “conformational switch” has been reported for the respiratory syncytial virus (RSV), where a single L305I substitution in the F protein modifies the neutralization epitopes (30). Why the best epitope landscape is not produced by the same VP2 sequence in two closely related species such as rainbow trout and Atlantic salmon is another interesting question that require more in-depth studies of their respective clonotypic responses after immunization with inactivated or live viral variants.

### Characteristics of the rainbow trout B cell response to the IPNV PT variant

Focusing on IPNV-PT immunised fish, our data provide a first description of the rainbow trout IgM response to this virus variant. A global analysis of repertoire diversity revealed that the B cell response in the spleen was stronger 2 months post-immunisation and had decreased significantly 2 months later. For most VH subgroups, the level of top clonotype sharing – a correlate of specific B cell response - was increased 2 months post-immunization compared to controls, while it was back to normal at 4 months post-immunization, except perhaps for VH2, VH4 and VH8. Thus, the modification of the spleen IgH repertoire decreased between 2 and 4 months post-immunisation, reflecting the decline in the number of spleen-activated B cells producing Abs. This overall decline was also observed in our previous work on the trout response against an attenuated strain of the rhabdovirus VHSV, where the acute response, which occurred 2–4 weeks post-immunisation and involved all VH subgroups, had almost entirely disappeared 5 months post-immunisation (22, 24).

Based on differential expression analysis, our data showed contrasting expression of VH subgroups VH1, VH4, and VH8 during the response to IPNV, especially for VJ combinations IGHV4D-24/J4D or J5D; IGHV4D-60/J1D; IGHV8-46/J4 or J11; IGHV1-21/J1 or J3, and IGHV8-30-40/J4. Although these gene combinations were consistently identified at both 2 and 4 months post-immunisation, the clonotypes involved in the response were different. Indeed, the most frequent clonotypes shared among individual fish 2 months post-immunisation were not among the top clonotypes shared 4 months post-immunisation. As our sharing analysis was based on an exact match, we checked whether mutations within CDR3 could explain this pattern; however, we failed to identify similar clonotypes that could mediate a public response present at both time points. This was in line with the reduced degree of sharing observed 4 months post-immunization.

Taken together, these observations suggest that public components are not dominant in the B cell response to IPNV. Also consistent with this notion is the lack of a leading public clonotype common to the responses to IPNV_PT (this work) and IPNV_TA (this work and (31)). This is in sharp contrast to the response to VHSV, for which large B cell expansions expressing public rearrangements were consistently found in all immunised fish, regardless of the protocol and sampling time (22, 24, 32). The observed lack of public antibody responses to IPNV is immunologically interesting, as it suggests a different mode of B cell activation and repertoire selection compared to VHSV. Of note, vaccination against VHSV using DNA or mRNA vaccines does not elicit a strong public response, as observed with the attenuated virus (33). Though, as long as protective immunity is achieved, the lack of public component in the response against IPNV does not pose a limitation for vaccine development or application.

The lack of overlap between rainbow trout responses to TA and PT variants is also in contrast to human B cell primary responses against influenza or SARS-CoV-2 viral variants, which generally show convergent responses with public components (34, 35).

### Interindividual variability

To standardise our comparisons between viral strains, we used a double-haploid line of rainbow trout, thereby avoiding genetic variation and reducing interindividual repertoire variability. All fish included in our experiments were approximately 15 months old, well past the fry stage, and highly susceptible to IPNV. Fish were immunised intraperitoneally with the same number of virus PFU from either IPNV_PT or IPNV_TA, and kept in separate tanks. Despite these measures taken to reduce the variability of responses within experimental groups, we observed a strong heterogeneity in the strength of B cell responses, as best evidenced by the IPNV-PT2 group, in which repertoire modifications were most prominent. This variation suggests that host responsiveness was heavily dependent on the immunological history of each fish, in line with a strong contribution of private components to the B cell response. These findings underscore that public antibody responses are not imposed by the host genetics or the antigen dose, but are shaped by the interplay between antigen structure, timing, and the immunological landscape of the host. For IPNV, this leads to diverse, private B cell responses, a hallmark of non-convergent immunity.

### Divergent responses and protection against new variants

Our work illustrates the strong impact of punctual variation in viral antigens on the composition of the rainbow trout B cell response against IPNV. An interesting question is to what extent such divergent B cell responses against IPNV variants may be correlated with a lack of Ab cross-protection against new variants after vaccination. The capacity of viruses to mutate their envelope proteins is central to escape from the host antibody memory response, as described for RSV (30). In the case of dengue virus E2 protein, immunization even imposes an increase of diversity to the IgH repertoire, leaving an individualized signature (36). Counteracting such subversion mechanisms, it has been shown in the mouse that newly recruited naïve B cells can dominate germinal centre responses after secondary exposure to the same virus or to a related variant (37, 38). Interestingly however, vaccination against SARS-coV2 led to more frequent expansions of B cell clones producing broadly specific Abs (*i.e.*, able to recognize different variants) in people who had been previously exposed to the infection than in naive individuals (39). Along the same line, B cells producing anti-influenza broadly neutralizing Abs are preferentially expanded after secondary exposure (40). While the dynamics of memory B cell responses and germinal centre reactions are likely different in fish and mammals, it will be interesting to see how a primary exposure to a IPNV variant could impact the B cell responses to secondary infections with other variants. The large difference between responses to TA and PT variant may correlate with mostly divergent secondary response or still allow efficient selection of broadly reactive clones.

## Conclusion

Our data provide the first comprehensive characterisation of the rainbow trout B cell response to two strains of IPNV, which differ in their host tropisms. A strong spleen IgM^+^ B cell response was induced only by the strain virulent to rainbow trout and involved all IGHV subgroups. No large and persistent public expansions could be found, in contrast to the response to VHSV. Our data demonstrate that viruses with minor differences in the VP2 protein elicit B cell responses with highly contrasting clonal structures and compositions. This divergence in clonal architecture suggests that even minor antigenic differences can shift the immunodominance landscape, altering how B cell repertoires are shaped during infection or vaccination. The absence of persistent public clonotypes limits the utility of IPNV-specific public antibodies as immunological biomarkers and underscores the need to evaluate protective immunity based on functional outcomes rather than sequence convergence. This study also has certain limitations, which primarily arise from (1) the focus on comparing IgHμ repertoires following immunization with these two IPNV variants, and (2) the use on isogenic fish as the experimental model. The kinetics of anti-IPNV Ab response and neutralizing titres induced by the TA and PT variants, and the detailed characterization of the viral infection will be important to provide a more complete overview. Using isogenic fish was essential to ensure a comparison of clonotypic responses between genetically similar fish but also limits our observations to a single genotype. Further experiments comparing rainbow trout responses to TA and PT IPNV will be important to extend our data and confirm the view that B cell responses to RNA viruses in fish are highly context-dependent, shaped by host genetics and by subtle viral features that determine antigen exposure and immunogenicity.

## Data Availability

The datasets presented in this study can be found in online repositories. The names of the repository/repositories and accession number(s) can be found below: https://www.ncbi.nlm.nih.gov/, PRJNA726017.
