# Supplementary material for: Contrasted modifications of IgM and IgT repertoires induced by high- and low-virulent infectious pancreatic necrosis virus strains in rainbow trout (Oncorhynchus mykiss)
Source: Front Immunol. 2026 Feb 4;16:1690504. doi: 10.3389/fimmu.2025.1690504 (PMC12913066; doi:10.3389/fimmu.2025.1690504)

Figure S1. Specific IgM response induced by immunisation WITH IPNV TA (A) and IPNV PT (B). IgM titers were determined using immunoassay (ELISA). «Controls» refers to the control group of non immunised fish, «pos» to serums previously tested and containing Antibodies against TA (A) or PT (B) IPNV, and «blank» to the condition without serum. ** $p<0.01$, * $p<0.05$

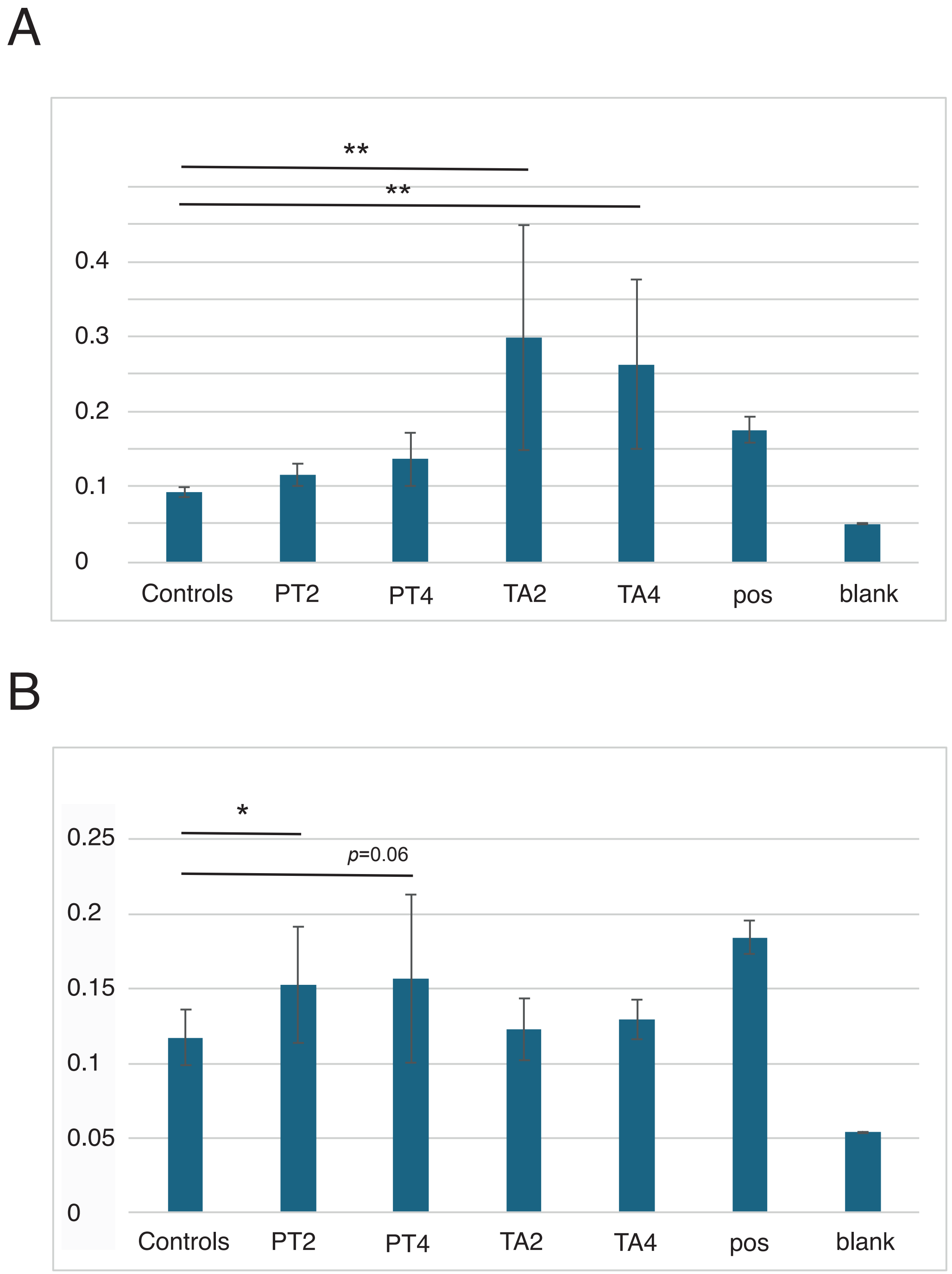

Supplement: Supplementary file 1 [file Image1.pdf]
