# Supplementary material for: Contrasted modifications of IgM and IgT repertoires induced by high- and low-virulent infectious pancreatic necrosis virus strains in rainbow trout (Oncorhynchus mykiss)
Source: Front Immunol. 2026 Feb 4;16:1690504. doi: 10.3389/fimmu.2025.1690504 (PMC12913066; doi:10.3389/fimmu.2025.1690504)

Figure S2. Clonotype frequency classified by decreasing rank, for each group A : IgM ; B : IgT.

The graph represents the distribution of clonotypic frequency ordered by decreasing rank of frequency within each experimental group. One dot may represent several clonotypes with the same MID count (ie, with the same frequency), for a given experimental group. For example, in panel A, only four dots represent the 9000 clonotypes ranked [1000-10000], because they were associated to only 4 different count values, hence to 4 distinct ranks. Color code is given on the right.

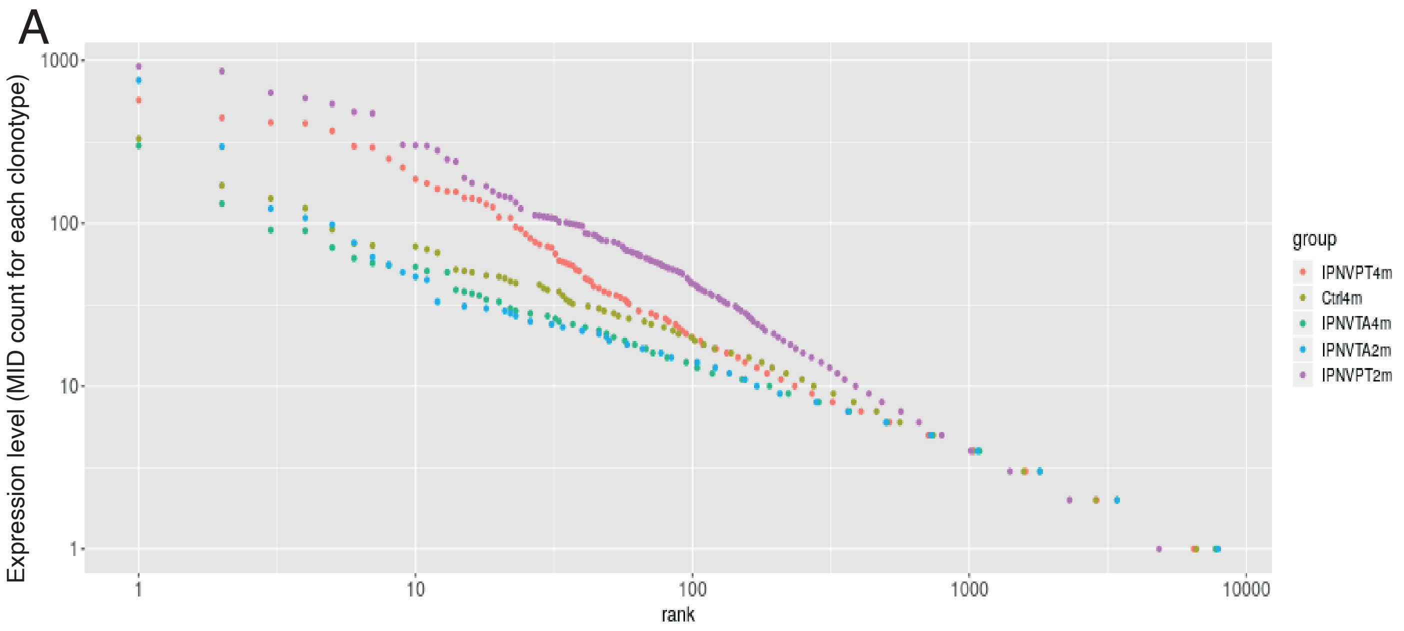

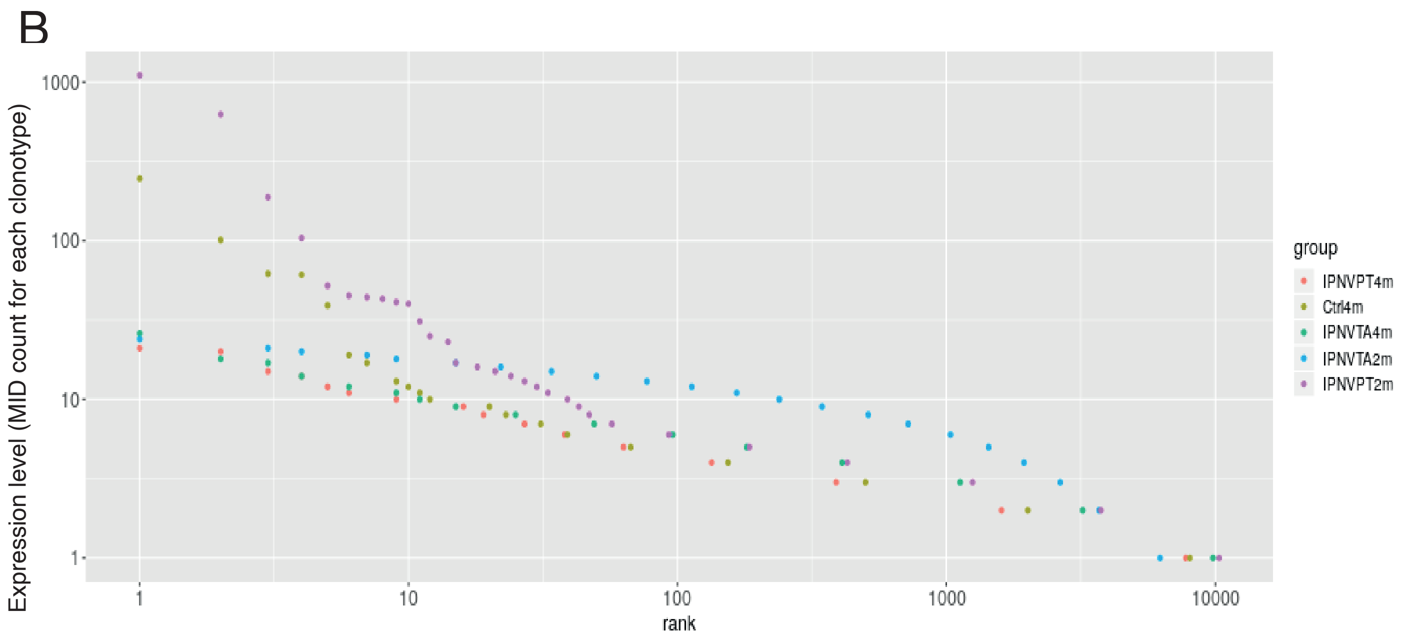

Supplement: Supplementary file 2 [file Image2.pdf]
