# Supplementary material for: Contrasted modifications of IgM and IgT repertoires induced by high- and low-virulent infectious pancreatic necrosis virus strains in rainbow trout (Oncorhynchus mykiss)
Source: Front Immunol. 2026 Feb 4;16:1690504. doi: 10.3389/fimmu.2025.1690504 (PMC12913066; doi:10.3389/fimmu.2025.1690504)

Figure S3. Differential analysis of expression of IGHV genes 2 months or 4 months for IgHμ after immunization with the TA virus. [X= log (FC); Y= log(p-value)].

VH genes were analyzed. Color codes are given at the bottom of each panel and refer to fold change or to significant p-value (5%). This analysis is based on data from four fish per group (subsampling of 12000 MID per fish).

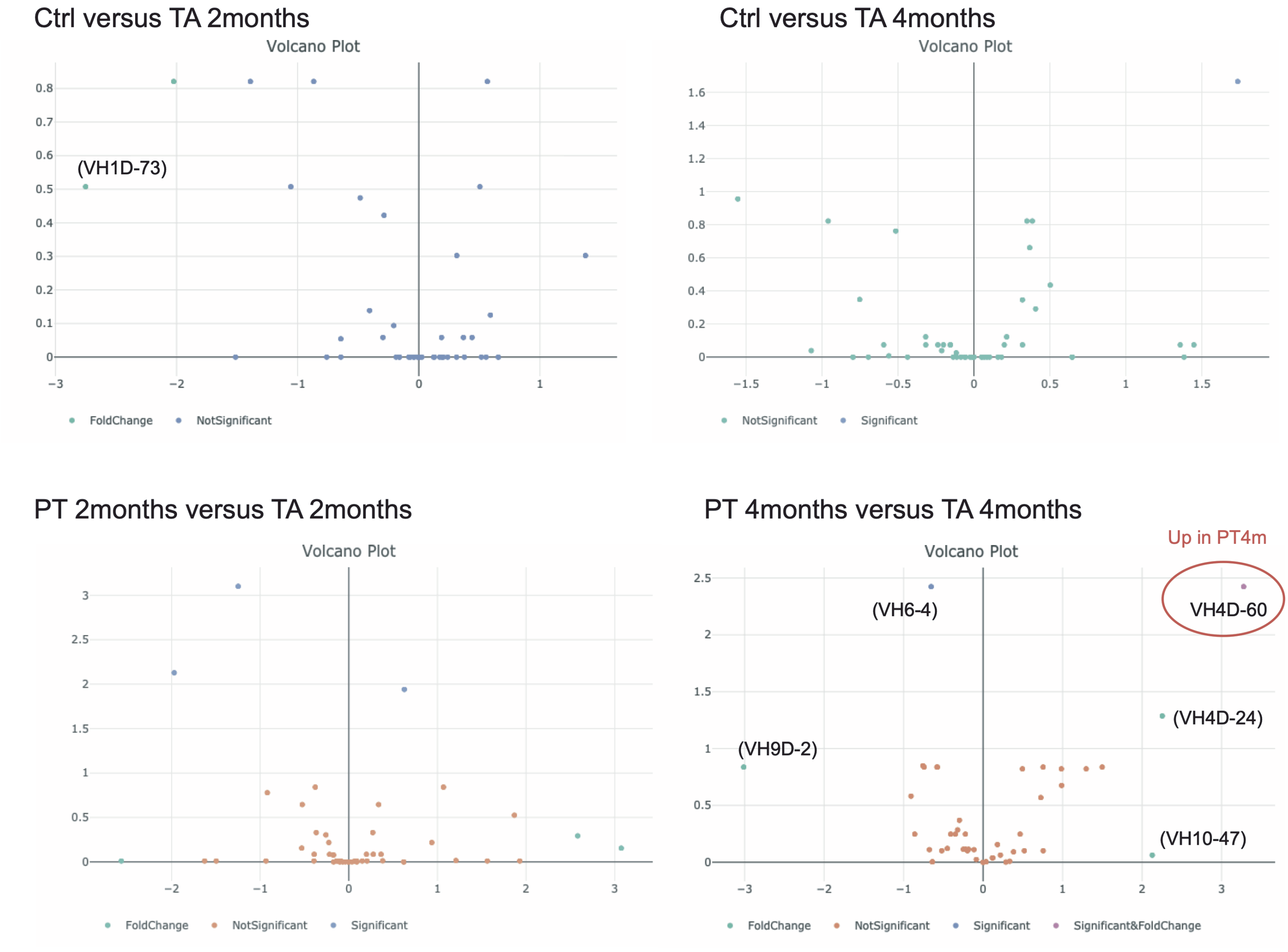

Supplement: Supplementary file 3 [file Image3.pdf]
