# Supplementary material for: Contrasted modifications of IgM and IgT repertoires induced by high- and low-virulent infectious pancreatic necrosis virus strains in rainbow trout (Oncorhynchus mykiss)
Source: Front Immunol. 2026 Feb 4;16:1690504. doi: 10.3389/fimmu.2025.1690504 (PMC12913066; doi:10.3389/fimmu.2025.1690504)

Figure S5. Heatmap of VH expression in IgM and IgT datasets

The expression was computed from 12000 MID subsamplings from each individual fish dataset. The number of each fish, and its experimental group is indicated at the bottom of each panel. Individual expression profiles were classified by hierarchical clustering (see bar at the top of each panel).

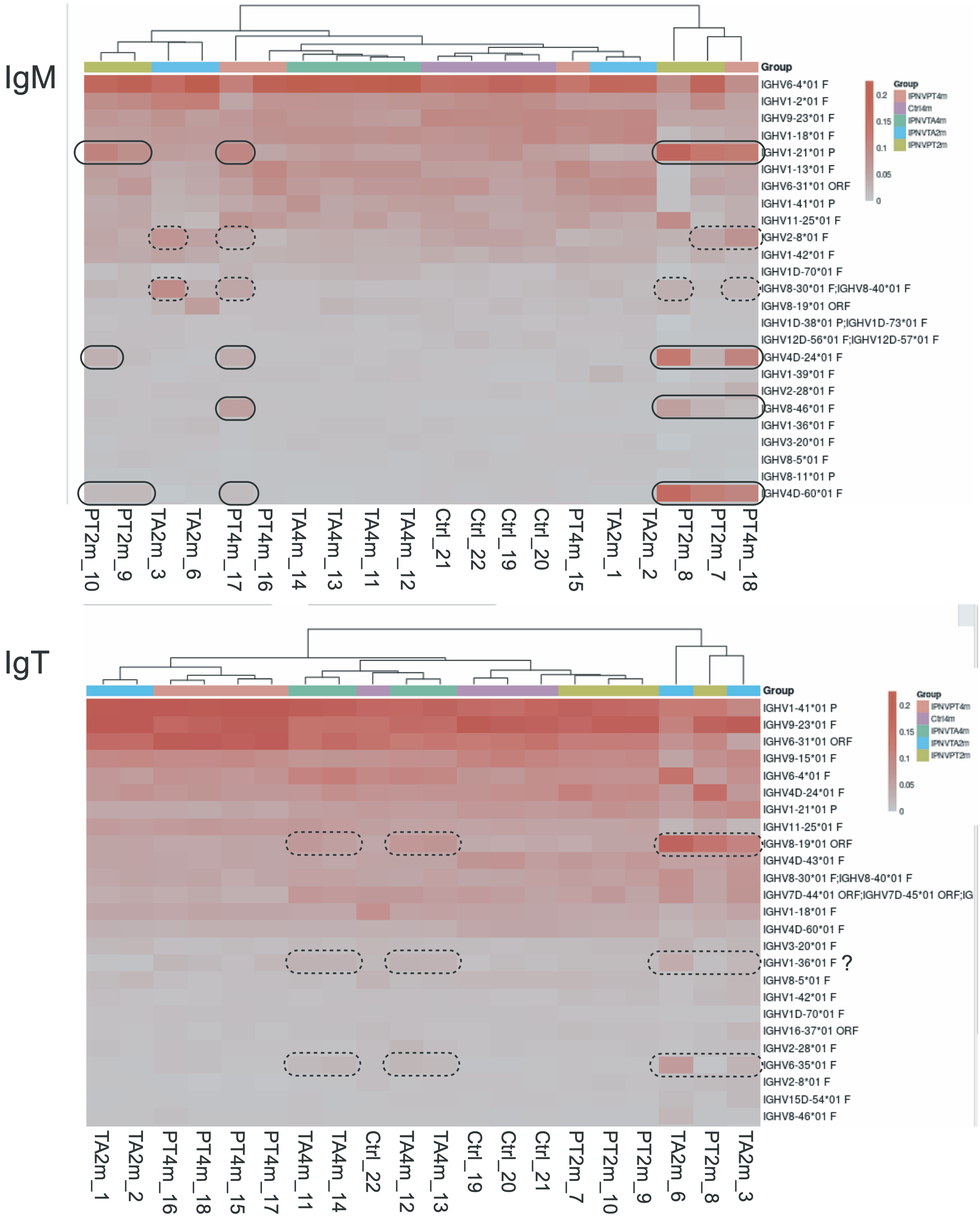

Supplement: Supplementary file 5 [file Image5.pdf]
