# Supplementary material for: Contrasted modifications of IgM and IgT repertoires induced by high- and low-virulent infectious pancreatic necrosis virus strains in rainbow trout (Oncorhynchus mykiss)
Source: Front Immunol. 2026 Feb 4;16:1690504. doi: 10.3389/fimmu.2025.1690504 (PMC12913066; doi:10.3389/fimmu.2025.1690504)

Figure S7

Cumulative expression of IgM Top50 clonotypes expressing a given VH subgroup shared by n individuals within control (Ctrl) and two groups of immunized fish (n=1, 2, 3 or 4). Lists ofTop50 clonotypes (TCL) are defined here *for a given VH subgroup*, within each analysed experimental group (Ctrl, first immunized group, second immunized group), as the non-redundant union of the lists of TOP50 clonotypes expressing a VH subgroup, computed from each 4 fish belonging to each group. The cumulative expression and sharing of clonotypes from the TCLCtlr reference list are represented in the left panels, while clonotypes from the TCL of the first or second immunized group are analysed in the middle and right panels respectively. Bar plots show the cumulated expression of TCL elements of each fish group, either among Ctrl (in blue), among the first immunized group (colour 1) or among the second immunized group (colour 2); in each small panel, bars noted 1, 2, 3 and 4 represent the cumulative expression of clonotypes found in only one fish or in 2, 3, or 4 fish, respectively. Bars are computed from the average values corresponding to top clonotypes found in 1–4 fish, over 10subsamplings of 10,000).

Panel A: IgM - Control group; 1st immunized group = IPNV TA2m; 2nd immunized group = IPNVPT2m
Colour code: ctrl=blue; IPNV TA2m : green (colour1); IPNV PT2m : red (colour2)

Panel B: IgT- Control group; 1st immunized group = IPNV TA2m; 2nd immunized group = IPNV
PT2m
Colour code: ctrl=blue; IPNV TA2m : green (colour1); IPNV PT4m : red (colour2)

Panel C: IgM - Control group; 1st immunized group = IPNV TA4m; 2nd immunized group = IPNV
PT4m
Colour code: ctrl=blue; IPNV TA2m : dark green (colour1); IPNV TA4m : dark red (colour2)

A. (IgM, Ctrl, TA2, PT2)

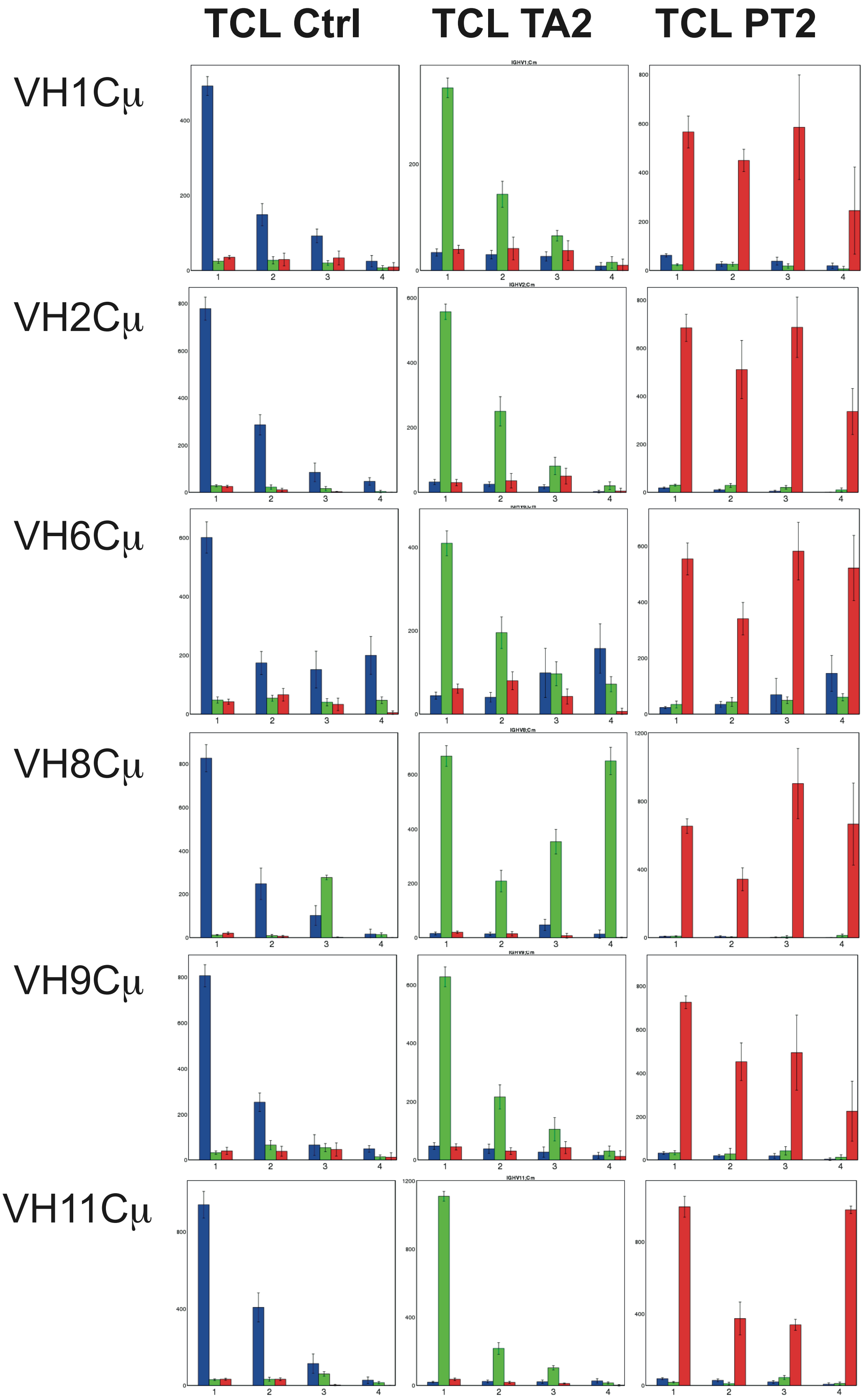

B. (IgT, CTRL, TA2, PT2)

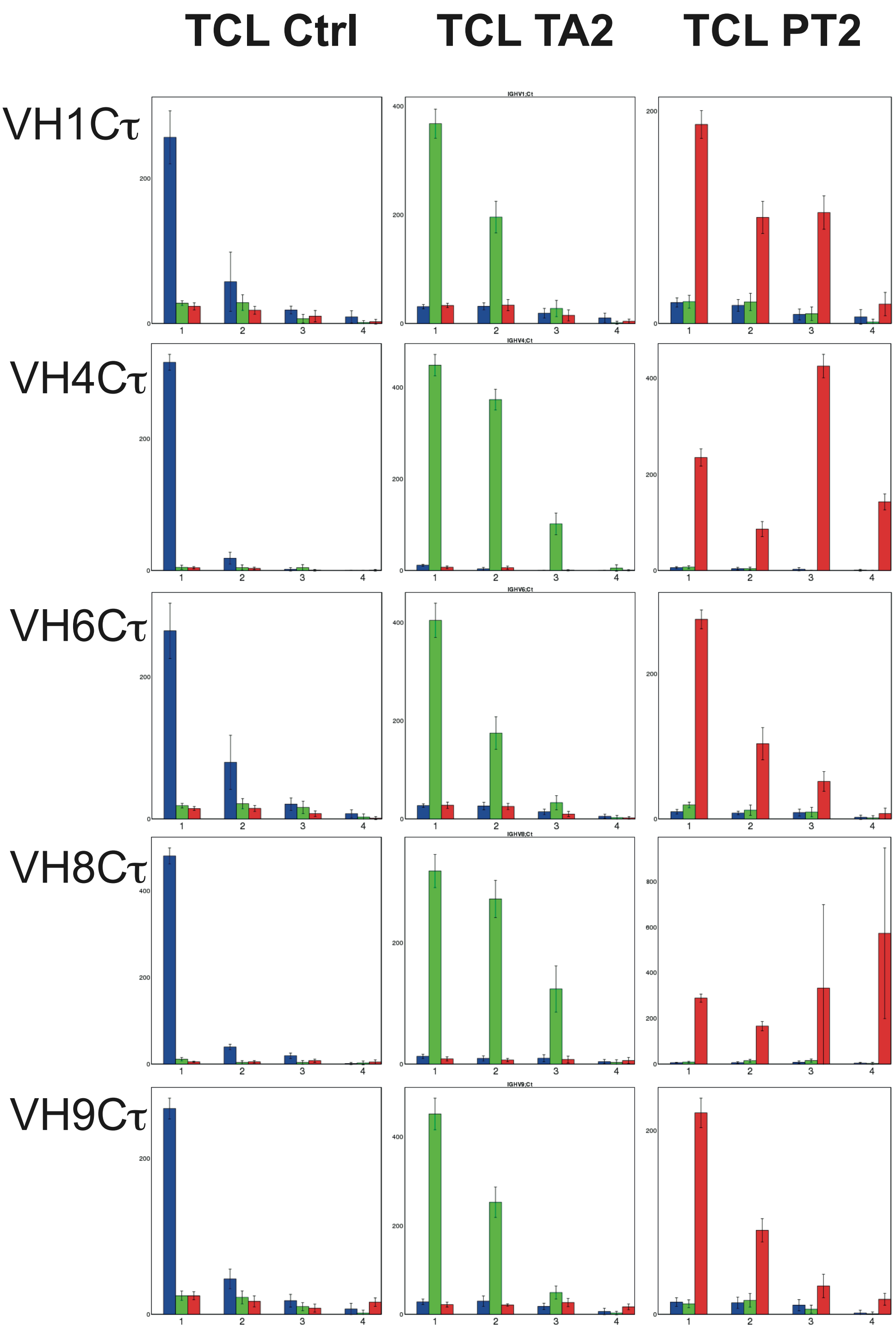

C. (IgM, Ctrl, TA4, PT4)

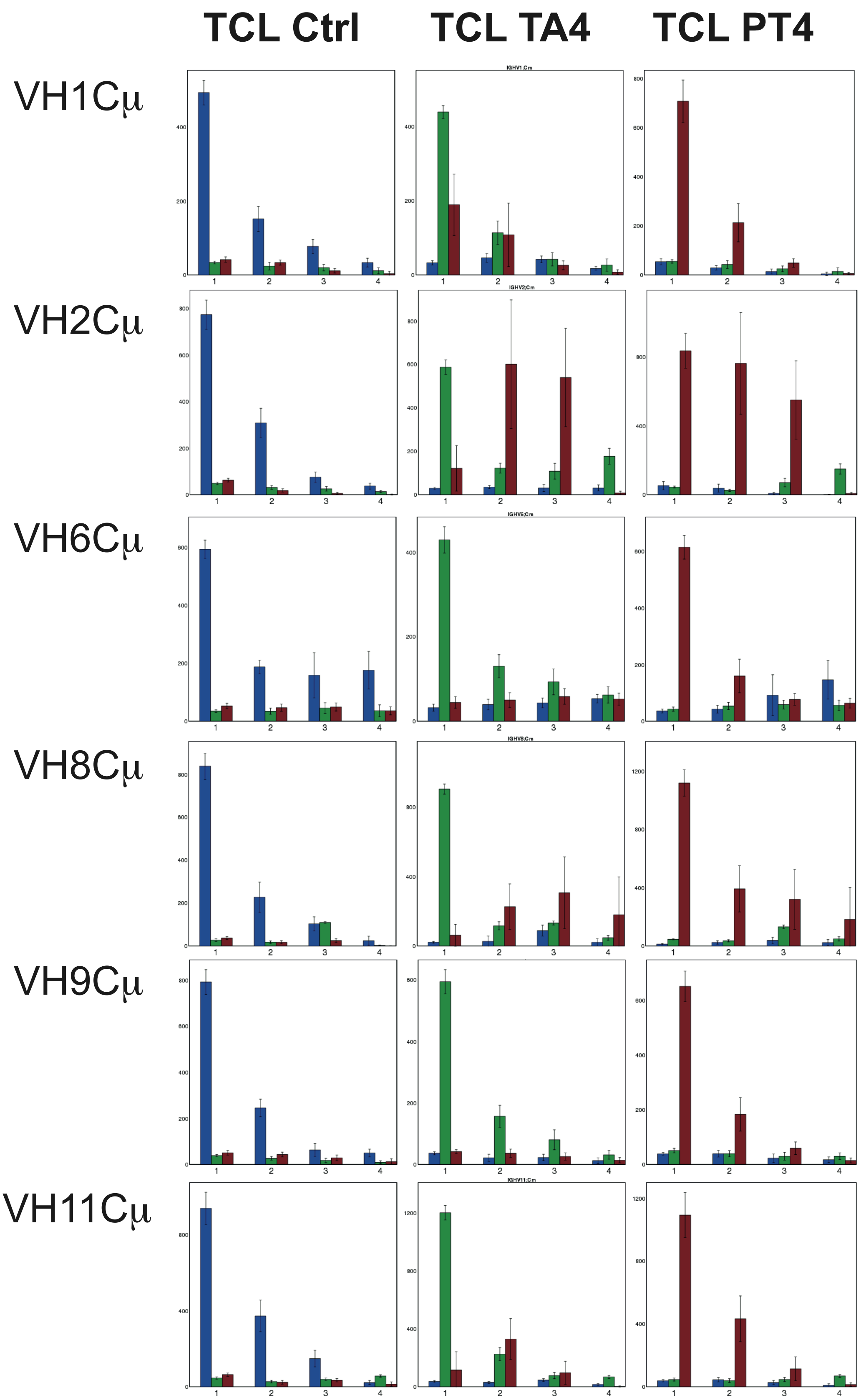

D. (IgT, CTRL, TA4, PT4)

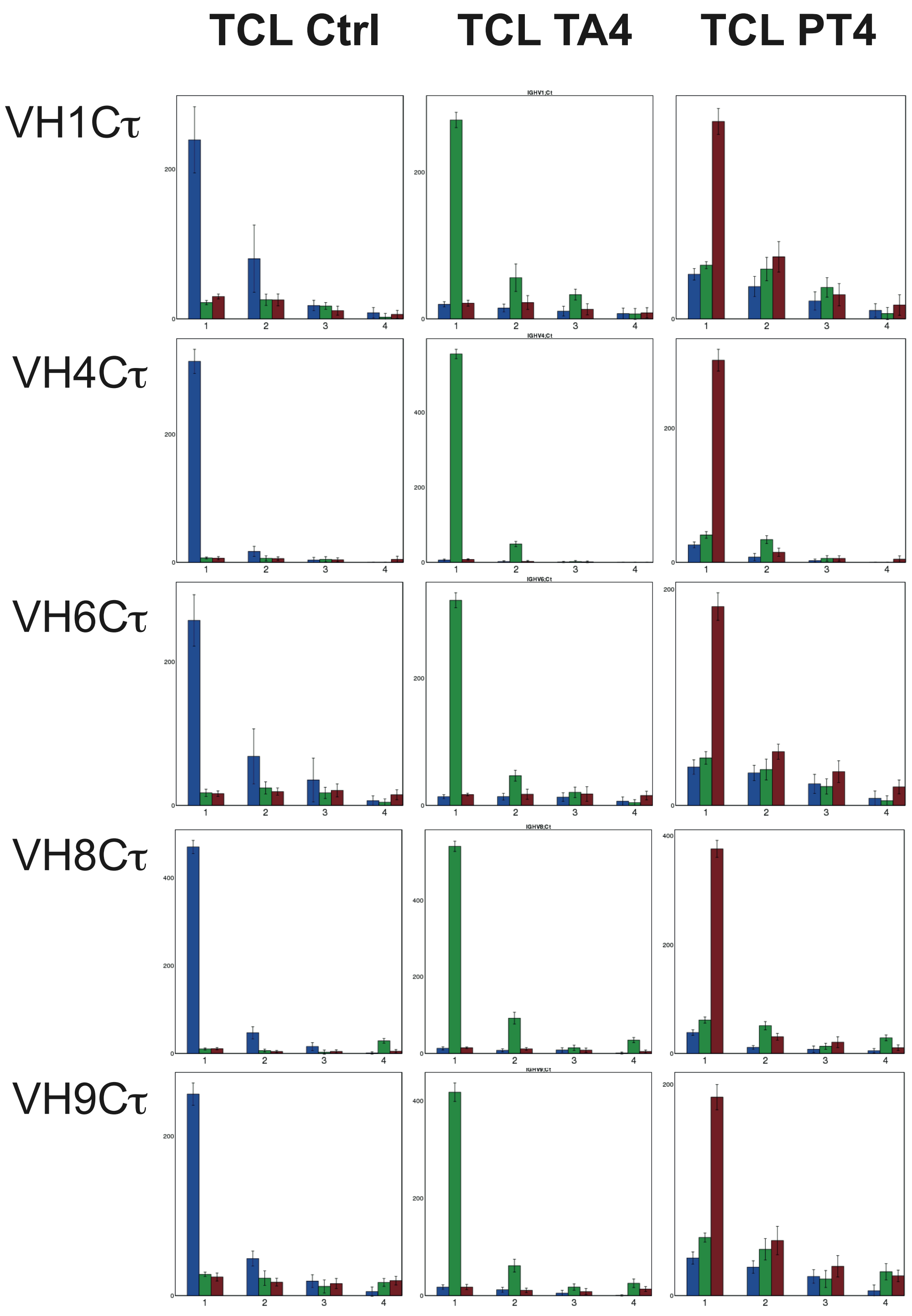

Supplement: Supplementary file 7 [file Image7.pdf]
