# Supplementary material for: Contrasted modifications of IgM and IgT repertoires induced by high- and low-virulent infectious pancreatic necrosis virus strains in rainbow trout (Oncorhynchus mykiss)
Source: Front Immunol. 2026 Feb 4;16:1690504. doi: 10.3389/fimmu.2025.1690504 (PMC12913066; doi:10.3389/fimmu.2025.1690504)

Figure S8.

Cumulative expression of IgM Top50 clonotypes expressing a given IGHV gene shared by nindividuals within control (Ctrl) and two groups of immunized fish. Lists of Top50 clonotypes(TCL) are defined here for a given IGHV, within each analyzed experimental group (Ctrl, firstimmunized group, second immunized group), as the non-redundant union of the lists of TOP50clonotypes expressing a IGHV, computed from each 4 fish belonging to each group. Thecumulative expression and sharing of clonotypes from the TCL_Ctlr reference list arerepresented in the left panels, while clonotypes from the TCL of the first or second immunizedgroup are analyzed in the middle and right panels respectively.

Bar plots show the cumulated expression of TCL elements of each fish group, either amongCtrl (in blue), among the first immunized group (colour 1) or among the second immunizedgroup (colour 2); in each small panel, bars noted 1, 2, 3 and 4 represent the cumulativeexpression of clonotypes found in only one fish or in 2, 3, or 4 fish, respectively. Bars arecomputed from the average values corresponding to top clonotypes found in 1–4 fish, over 10subsamplings of 10,000).

Panel A1: IgM - Control group; 1st immunized group = IPNV PT2m; 2nd immunized group = IPNV PT4m
Colour code: ctrl=blue; IPNV PT2m : red (colour1); IPNV PT4m : dark red (colour2)
Panel A2: IgT- Control group; 1st immunized group = IPNV PT2m; 2nd immunized group = IPNV PT4m
Colour code: ctrl=blue; IPNV PT2m : red (colour1); IPNV PT4m : dark red (colour2)
Panel B1: IgM - Control group; 1st immunized group = IPNV TA2m; 2nd immunized group = IPNV TA4m
Colour code: ctrl=blue; IPNV TA2m : green (colour1); IPNV TA4m : dark green (colour2)
Panel B2: IgT - Control group; 1st immunized group = IPNV TA2m; 2nd immunized group = IPNV TA4m
Colour code: ctrl=blue; IPNV TA2m : green (colour1); IPNV TA4m : dark green (colour2)
Panel C: IGM - Control group; 1st immunized group = IPNV TA2m; 2nd immunized group = IPNV PT2m
Colour code: ctrl=blue; IPNV PT2m : red (colour1); IPNV TA2m : green (colour2)
Blue circles and orange arrows denote remarkable sharing.

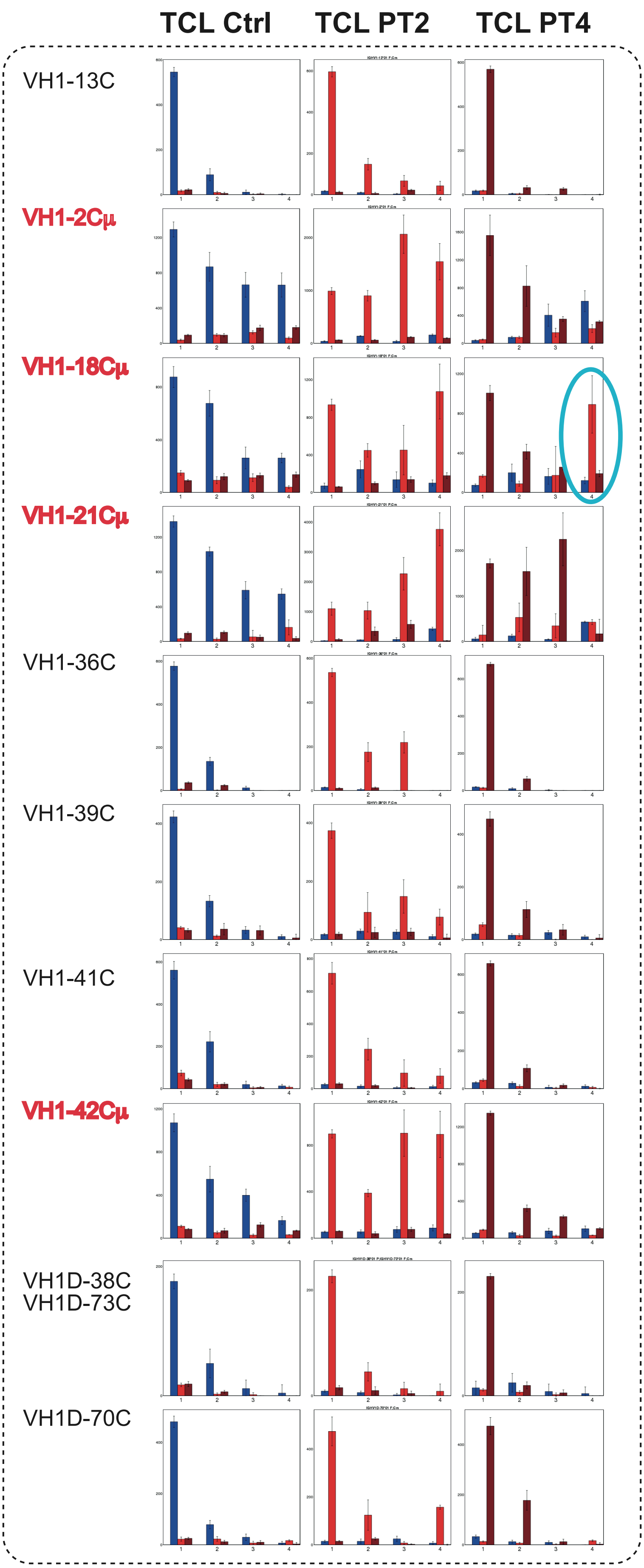

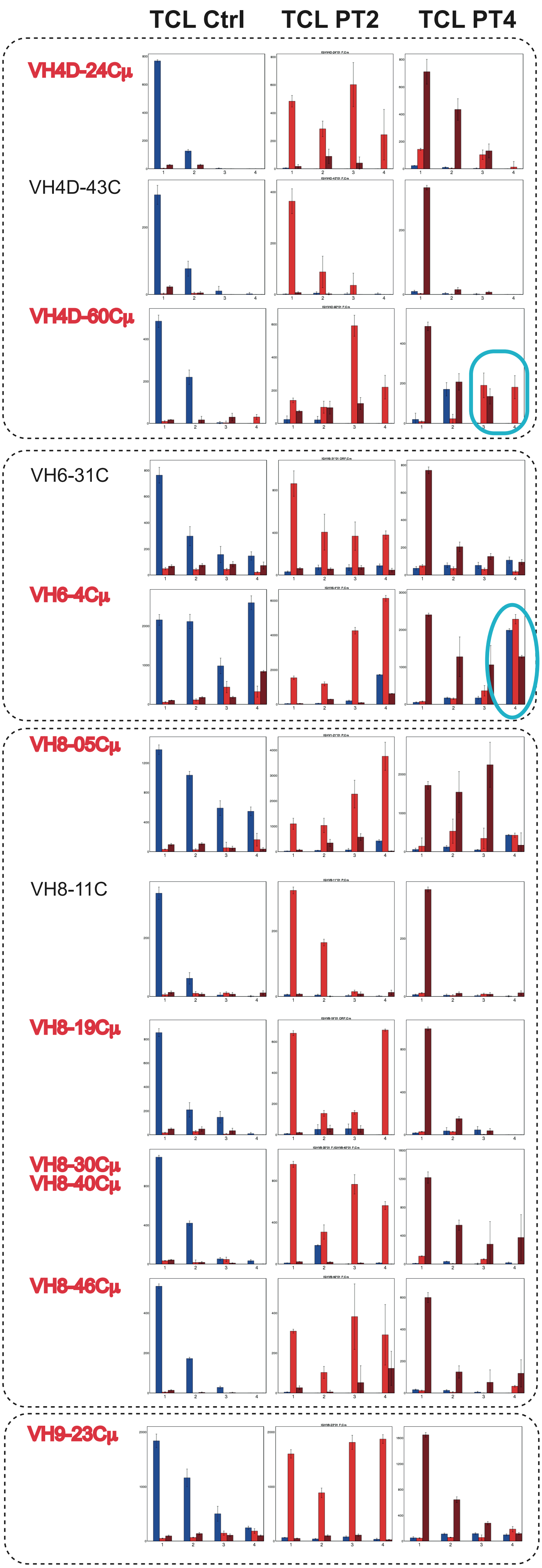

Figure S7

A1. IgM, CTRL, PT2, PT4

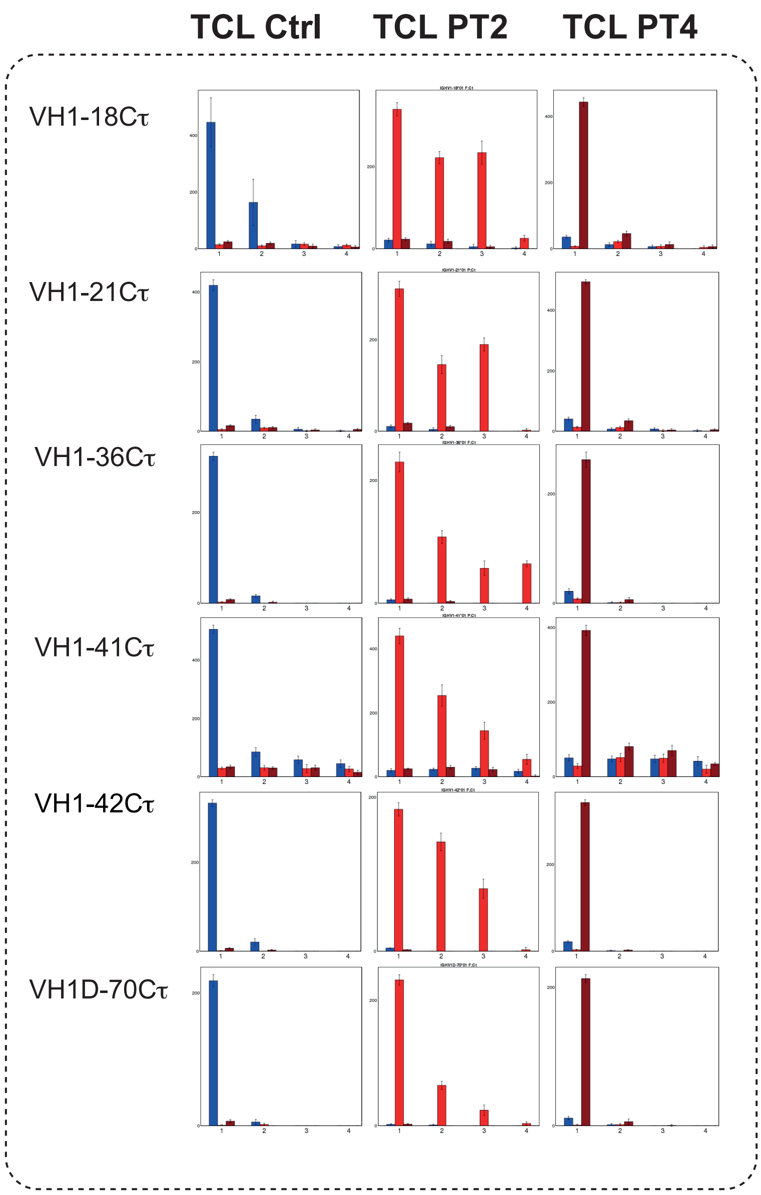

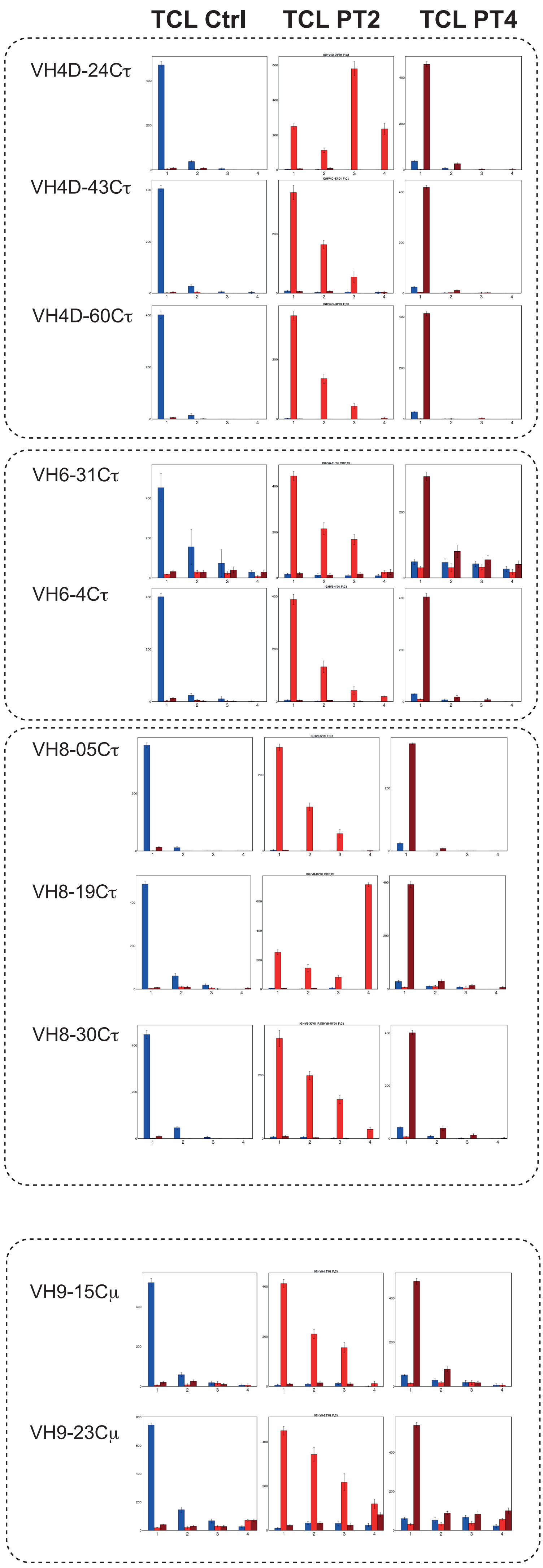

A2. IgT, CTRL, PT2, PT4

B1. IgM, CTRL, TA2, TA4

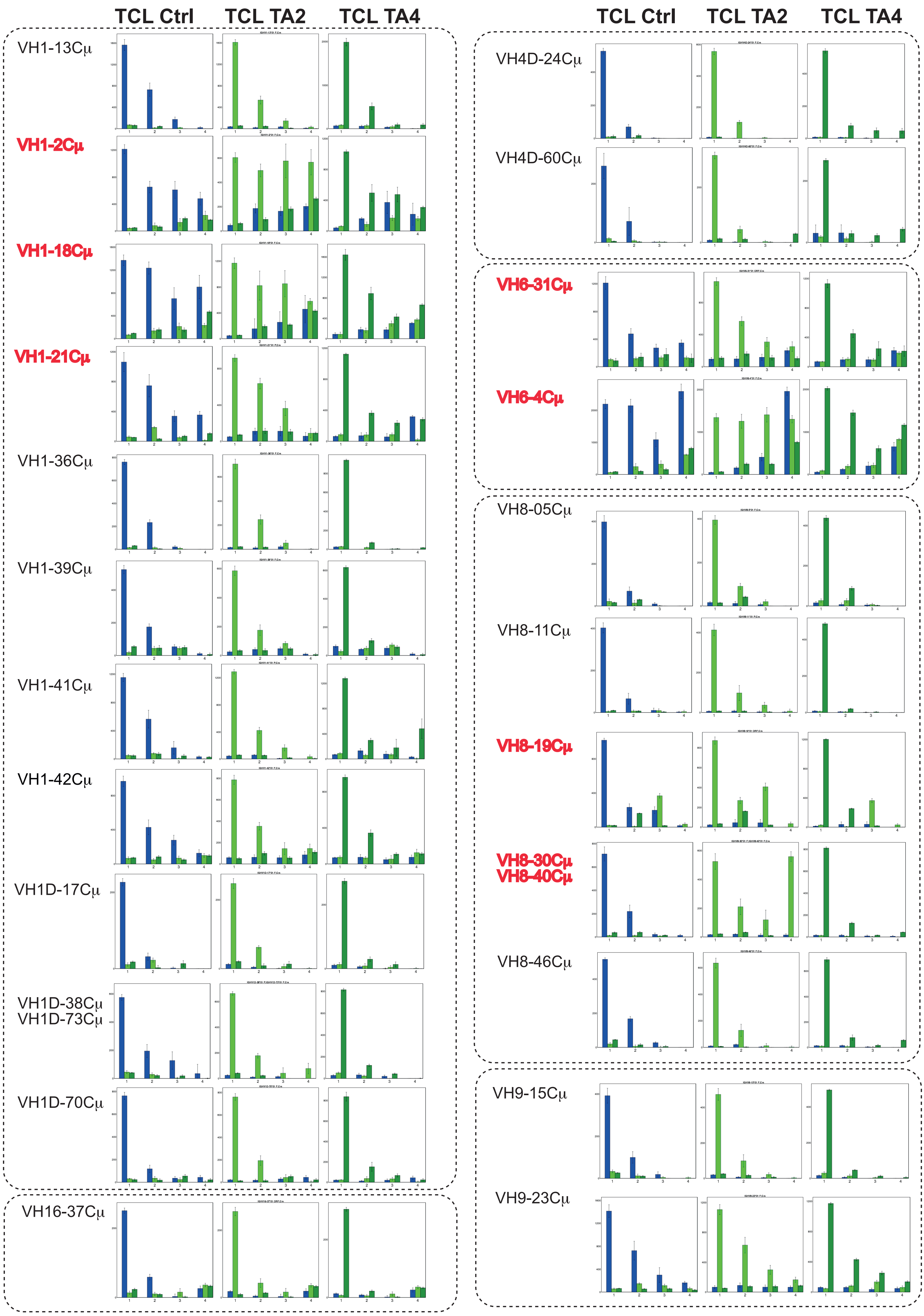

B2. IgT CTRL, TA2, TA4

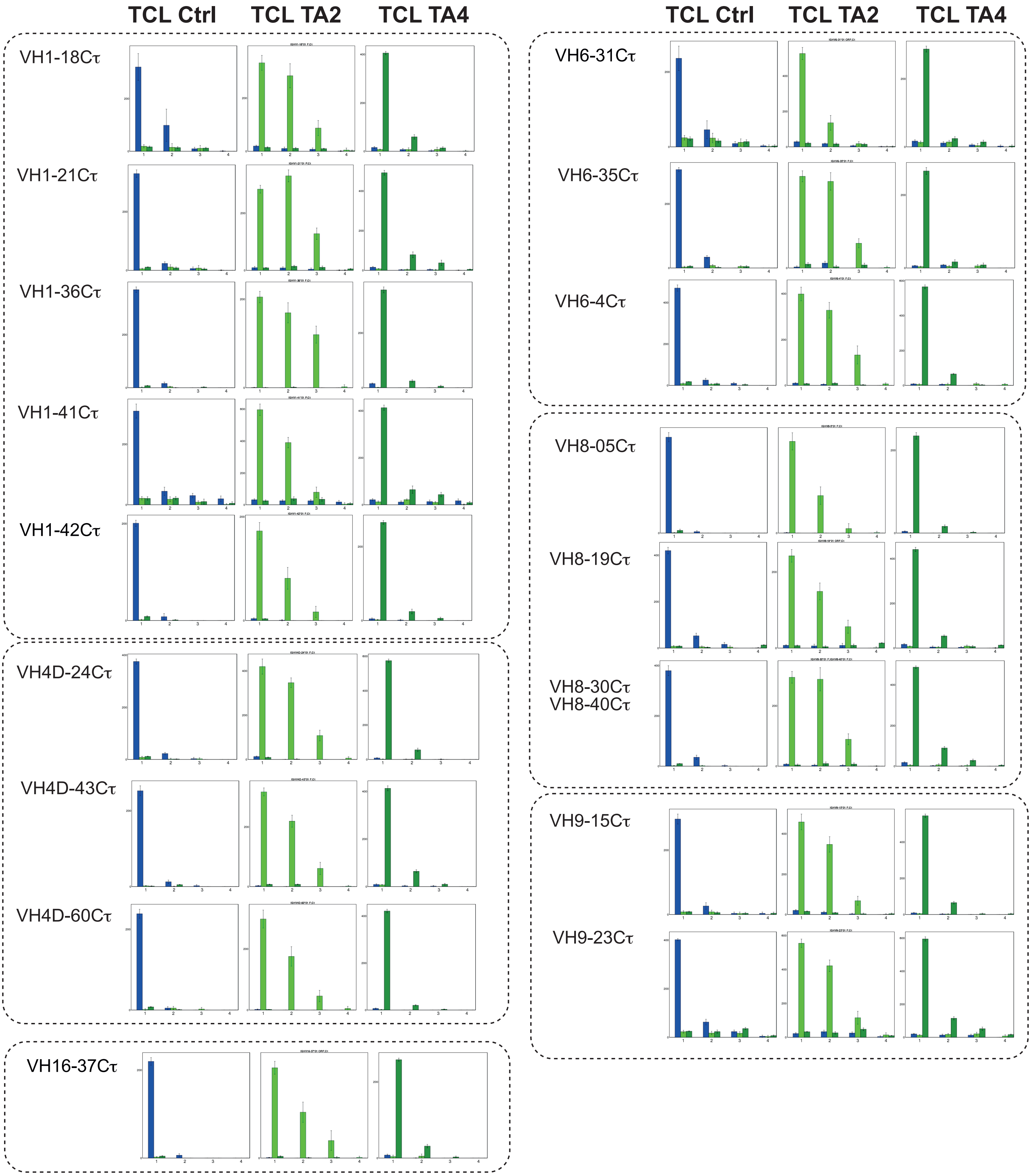

C. IgM , Ctrl, TA2, PT2

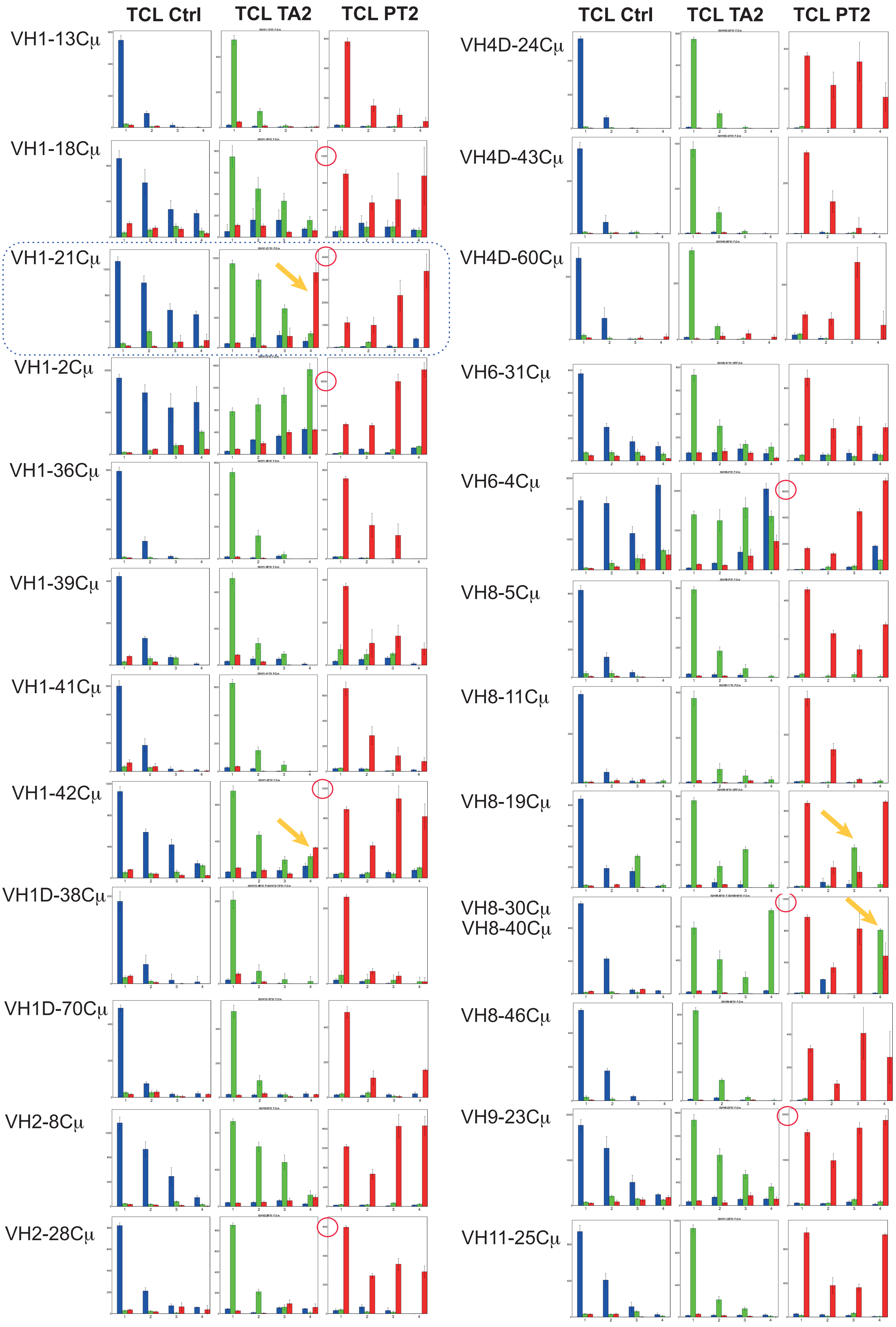

Supplement: Supplementary file 8 [file Image8.pdf]
