# Supplementary figures and images for: Contrasted modifications of IgM and IgT repertoires induced by high- and low-virulent infectious pancreatic necrosis virus strains in rainbow trout (Oncorhynchus mykiss)
Source: Front Immunol. 2026 Feb 4;16:1690504. doi: 10.3389/fimmu.2025.1690504 (PMC12913066; doi:10.3389/fimmu.2025.1690504)

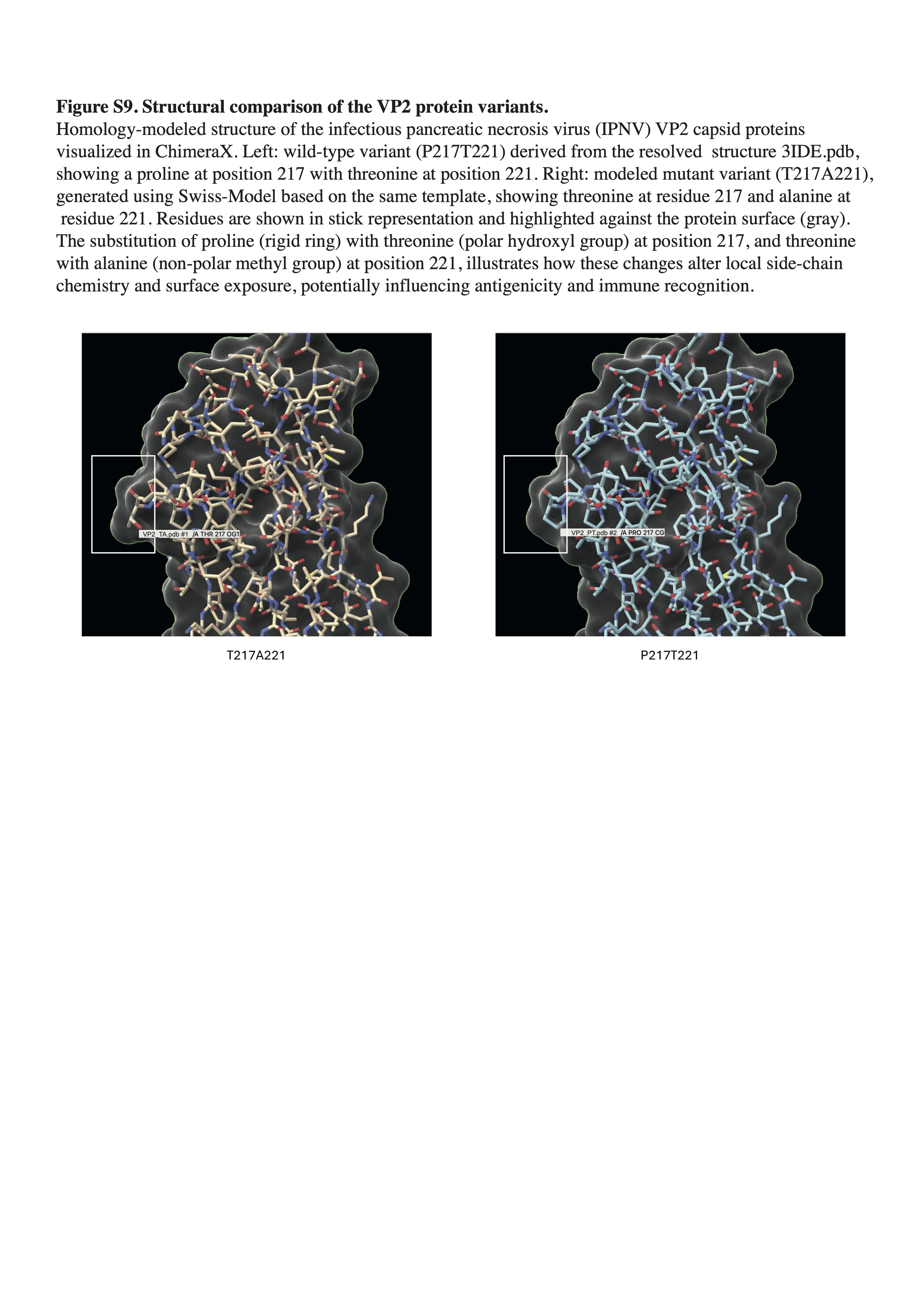

Supplement: Supplementary file 9 [file Image9.jpeg]
