# Supplementary material for: Contrasted modifications of IgM and IgT repertoires induced by high- and low-virulent infectious pancreatic necrosis virus strains in rainbow trout (Oncorhynchus mykiss)
Source: Front Immunol. 2026 Feb 4;16:1690504. doi: 10.3389/fimmu.2025.1690504 (PMC12913066; doi:10.3389/fimmu.2025.1690504)
Supplement: Supplementary file 10 [file Table1.pdf]

Table S1. Primers used in this study.

Primers	Sequences 5’-> 3’
Cmu1	CACATTGCGCAAGAGGGAACAA
Cmu2	AGAGACGGCTGCTGCAGATATTCC
Ctau	GTTCCACAGTTCATAAGAGTG
Rd1_2N_Cmu1	AATGATACGGCGACCACCGAGATCTACACTCTTTCCCTACACGACGCTCTTCCGATCTTTGCGCAAGAGGGAACAAAGTC
Rd1_2N-Ctau	AATGATACGGCGACCACCGAGATCTACACTCTTTCCCTACACGACGCTCTTCCGATCTACAGTTCATAAGAGTGAGTAG
Rd1_4N_Cmu1	AATGATACGGCGACCACCGAGATCTACACTCTTTCCCTACACGACGCTCTTCCGATCTCATTGCGCAAGAGGGAACAAAG
Rd1_4N-Ctau	AATGATACGGCGACCACCGAGATCTACACTCTTTCCCTACACGACGCTCTTCCGATCTTTACAGTTCATAAGAGTGAGTAG
Rd2_FBD1_Rd2p	CAAGCAGAAGACGGCATACGAGATCGTGATGTGACTGGAGTTCAGACGTGTGCTCTTCCGATCT
Rd2_FBD10_Rd2p	CAAGCAGAAGACGGCATACGAGATAAGCTAGTGACTGGAGTTCAGACGTGTGCTCTTCCGATCT
Rd2_FBD11_Rd2p	CAAGCAGAAGACGGCATACGAGATGTAGCCGTGACTGGAGTTCAGACGTGTGCTCTTCCGATCT
Rd2_FBD12_Rd2p	CAAGCAGAAGACGGCATACGAGATTACAAGGTGACTGGAGTTCAGACGTGTGCTCTTCCGATCT
Rd2_FBD13_Rd2p	CAAGCAGAAGACGGCATACGAGATTTGACTGTGACTGGAGTTCAGACGTGTGCTCTTCCGATCT
Rd2_FBD14_Rd2p	CAAGCAGAAGACGGCATACGAGATGGAACTGTGACTGGAGTTCAGACGTGTGCTCTTCCGATCT
Rd2_FBD15_Rd2p	CAAGCAGAAGACGGCATACGAGATTGACATGTGACTGGAGTTCAGACGTGTGCTCTTCCGATCT
Rd2_FBD16_Rd2p	CAAGCAGAAGACGGCATACGAGATGGACGGGTGACTGGAGTTCAGACGTGTGCTCTTCCGATCT
Rd2_FBD2_Rd2p	CAAGCAGAAGACGGCATACGAGATACATCGGTGACTGGAGTTCAGACGTGTGCTCTTCCGATCT
Rd2_FBD22_Rd2p	CAAGCAGAAGACGGCATACGAGATCGTACGGTGACTGGAGTTCAGACGTGTGCTCTTCCGATCT
Rd2_FBD25_Rd2p	CAAGCAGAAGACGGCATACGAGATATCAGTGTGACTGGAGTTCAGACGTGTGCTCTTCCGATCT
Rd2_FBD3_Rd2p	CAAGCAGAAGACGGCATACGAGATGCCTAAGTGACTGGAGTTCAGACGTGTGCTCTTCCGATCT
Rd2_FBD4_Rd2p	CAAGCAGAAGACGGCATACGAGATTGGTCAGTGACTGGAGTTCAGACGTGTGCTCTTCCGATCT
Rd2_FBD5_Rd2p	CAAGCAGAAGACGGCATACGAGATCACTGTGTGACTGGAGTTCAGACGTGTGCTCTTCCGATCT
Rd2_FBD6_Rd2p	CAAGCAGAAGACGGCATACGAGATATTGGCGTGACTGGAGTTCAGACGTGTGCTCTTCCGATCT
Rd2_FBD9_Rd2p	CAAGCAGAAGACGGCATACGAGATCTGATCGTGACTGGAGTTCAGACGTGTGCTCTTCCGATCT
Rd2p_UMI_5RACE	GTGACTGGAGTTCAGACGTGTGCTCTTCCGATCTNNNNNNNNNNNNNNNNAAGCAGTGGTATCAACGCAGAGT
