# Supplementary material for: Contrasted modifications of IgM and IgT repertoires induced by high- and low-virulent infectious pancreatic necrosis virus strains in rainbow trout (Oncorhynchus mykiss)
Source: Front Immunol. 2026 Feb 4;16:1690504. doi: 10.3389/fimmu.2025.1690504 (PMC12913066; doi:10.3389/fimmu.2025.1690504)

Table S2. IGHV usage in Ig repertoire of control fish

IGHV annotation from IMGT VQUEST	Cummulated expression in fish from the control group
IGHV6-4*01 F	7947
IGHV9-45*01 F	4711
IGHV1-2*01 F	4168
IGHV1-18*01 F	4036
IGHV1-21*01 P	3294
IGHV6-31*01 ORF	2991
IGHV1-13*01 F	2859
IGHV1-41*01 P	2492
IGHV11-25*01 F	2384
IGHV2-8*01 F	2330
IGHV1-42*01 F	1698
IGHV1D-70*01 F	771
IGHV8-30*01 F;IGHV8-40*01 F	755
IGHV8-19*01 ORF	742
IGHV12D-56*01 F;IGHV12D-57*01	737
IGHV1D-38*01 P;IGHV1D-73*01 F	622
IGHV2-28*01 F	535
IGHV8-5*01 F	429
IGHV4D-24*01 F	425
IGHV1-36*01 F	420
IGHV4D-60*01 F	406
IGHV8-11*01 P	398
IGHV3-20*01 F	380
IGHV1-39*01 F	358
IGHV9-15*01 F	353
IGHV8-46*01 F	269
IGHV4D-43*01 F	234
IGHV15D-54*01 F	227
IGHV7D-44*01 ORF;IGHV7D-45*01	226
IGHV1D-17*01 F	214
IGHV16-37*01 ORF	142
IGHV6D-76*01 F	124
IGHV15D-49*01 P	105
IGHV1D-38*01 P	85
IGHV9D-2*01 F	30
IGHV12D-36*01 F	26
IGHV15D-49*01 P;IGHV15D-69*01	22
IGHV2D-50*01 P;IGHV2D-51*01 F;I	17
IGHV6-35*01 F	15
IGHV1D-15*01 ORF	12
IGHV14-33*01 P	4
IGHV1D-73*01 F	2
IGHV6D-40*01 F	2
IGHV16D-79*01 P	1
IGHV1D-35*01 F	1
IGHV9D-2*01 F	1
total	48000
top 5 IGHV	24156
Proportion	0,50325

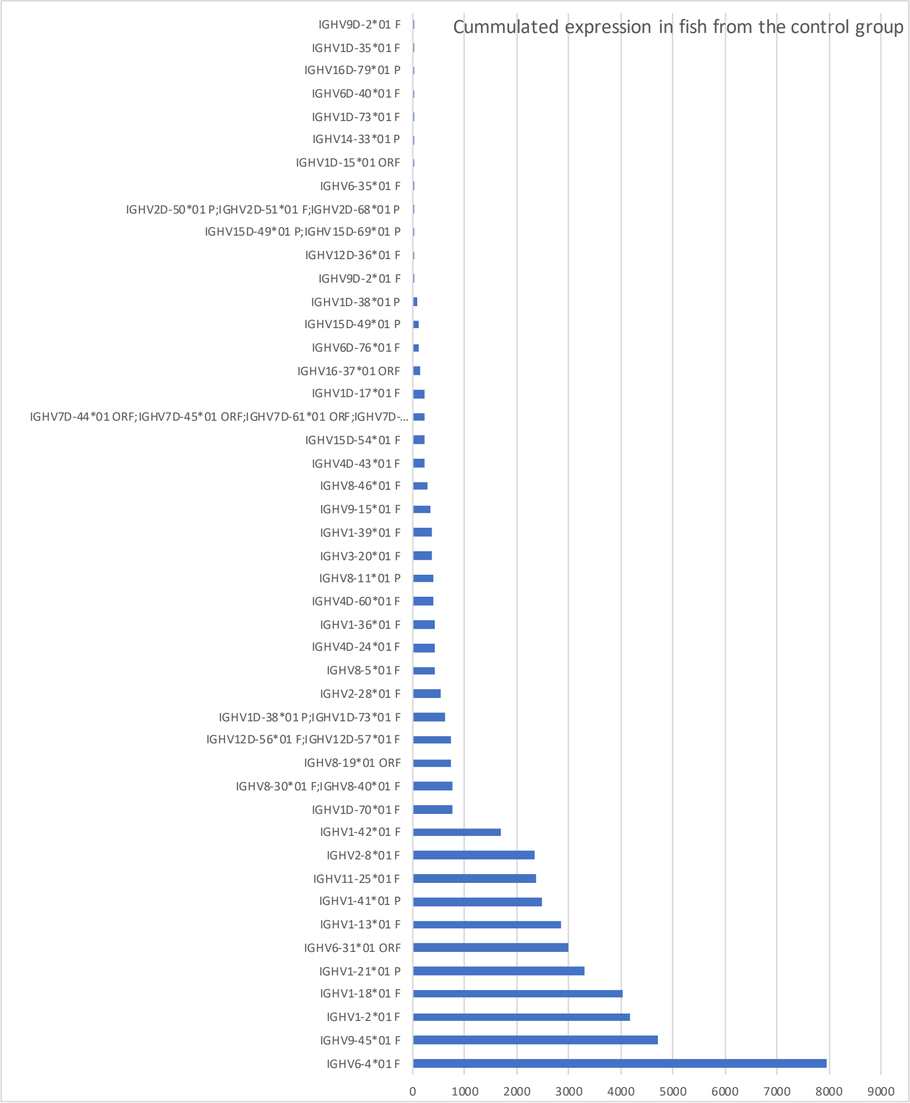

Supplement: Supplementary file 11 [file Table2.pdf]
